# High-frequency spinal cord stimulation at 10 kHz for the treatment of painful diabetic neuropathy: design of a multicenter, randomized controlled trial (SENZA-PDN)

**DOI:** 10.1186/s13063-019-4007-y

**Published:** 2020-01-15

**Authors:** Nagy A. Mekhail, Charles E. Argoff, Rod S. Taylor, Christian Nasr, David L. Caraway, Bradford E. Gliner, Jeyakumar Subbaroyan, Elizabeth S. Brooks

**Affiliations:** 1 Evidence-Based Pain Management Research, Cleveland Clinic, C25, 9500 Euclid Avenue, Cleveland, OH 44195 USA; 2 Department of Neurology, Albany Medical College, MC 70, 47 New Scotland Avenue, Albany, NY 12208 USA; 3 Institute of Health and Well Being, University of Glasgow, 1 Lilybank Gardens, Glasgow G12 8RZ, Scotland, UK; 4 College of Medicine and Health, University of Exeter, St. Luke’s Campus, Heavitree Road, Exeter EX1 2LU, England, UK; 5 Department of Endocrinology & Metabolism, Cleveland Clinic, F20, 9500 Euclid Avenue, Cleveland, OH 44195 USA; 6 Nevro Corp, 1800 Bridge Parkway, Redwood City, CA 94065 USA

## Abstract

**Background:**

Painful diabetic neuropathy (PDN), a debilitating and progressive chronic pain condition that significantly impacts quality of life, is one of the common complications seen with long-standing diabetes mellitus. Neither pharmacological treatments nor low-frequency spinal cord stimulation (SCS) has provided significant and long-term pain relief for patients with PDN. This study aims to document the value of 10-kHz SCS in addition to conventional medical management (CMM) compared with CMM alone in patients with refractory PDN.

**Methods:**

In a prospective, multicenter, randomized controlled trial (SENZA-PDN), 216 subjects with PDN will be assigned 1:1 to receive 10-kHz SCS combined with CMM or CMM alone after appropriate institutional review board approvals and followed for 24 months. Key inclusion criteria include (1) symptoms of PDN for at least 12 months, (2) average pain intensity of at least 5 cm—on a 0- to 10-cm visual analog scale (VAS)—in the lower limbs, and (3) an appropriate candidate for SCS. Key exclusion criteria include (1) large or gangrenous ulcers or (2) average pain intensity of at least 3 cm on VAS in the upper limbs or both. Along with pain VAS, neurological assessments, health-related quality of life, sleep quality, and patient satisfaction will be captured. The primary endpoint comparing responder rates (≥50% pain relief) and safety rates between the treatment groups will be assessed at 3 months. Several secondary endpoints will also be reported on.

**Discussion:**

Enrollment commenced in 2017 and was completed in 2019. This study will help to determine whether 10-kHz SCS improves clinical outcomes and health-related quality of life and is a cost-effective treatment for PDN that is refractory to CMM.

**Trial registration:**

ClincalTrials.gov identifier: NCT03228420 (registered 24 July 2017).

## Background

Globally, 422 million people have diabetes, resulting in USD $1.7 trillion in direct and indirect costs [1]. According to data from the Centers for Disease Control and Prevention, 29 million people in the US are currently living with diabetes and another 86 million with prediabetes, resulting in $245 billion in health-care costs and lost productivity [2]. About 20% of patients with diabetes will develop painful diabetic neuropathy (PDN) [3], a debilitating and progressive chronic pain condition that significantly impacts quality of life.

Peripheral neuropathy from damage to peripheral nerves may result in pain, numbness, or weakness (or a combination of these) in the affected limb. Damage may affect small (myelinated Aδ and unmyelinated C) fibers along with injury to large myelinated fibers. One of the classifications for peripheral neuropathy is based on whether the damage is to a single nerve (mononeuropathy) or multiple nerves (polyneuropathy). The causes of polyneuropathy may include metabolic (e.g., chronic renal failure), endocrine disorders (e.g., PDN), treatment-induced toxicity (e.g., radiation, chemotherapy, or alcohol-induced neuropathy), infection (Lyme disease and post-herpetic neuralgia caused by herpes zoster virus), autoimmune disorders (Guillain–Barré syndrome and Charcot–Marie–Tooth neuropathy), compression (carpal tunnel syndrome, tarsal tunnel syndrome, ulnar neuropathy, and peroneal neuropathy), and trauma (trauma-induced neuropathy). Nearly half of cases of peripheral neuropathy are diagnosed as idiopathic [4].

The American Chronic Pain Association estimates that more than 15 million people in the US and Europe have some degree of neuropathic pain. More than 2 in 100 persons are estimated to have peripheral neuropathy; the incidence rises to 8 in 100 for those who are 55 or older [5]. In Europe, the prevalence of PDN ranged from 5.8% to 34.0% [6]. The incidences of PDN were reported to be 0.72 per 1000 persons per year in the Netherlands [7] and 0.64–0.69 per 1000 persons per year in the UK [8]. PDN is very taxing to the individual patient because of pain, impaired quality of life, and increased disability [9, 10] and to society as a whole because of the significant impact on the workforce and the increased cost of health care [11, 12]

Anticonvulsant medications, including gabapentin and pregabalin, are among the most commonly prescribed medications for neuropathic pain due to PDN [13]. Pregabalin, or (*S*)-3-(aminomethyl)-5-methylhexanoic acid, is an analog of the inhibitory neurotransmitter gamma-aminobutyric acid (GABA). It is a compound that acts on the central nervous system, producing analgesic, anticonvulsant, and anxiolytic effects. Clinical studies have demonstrated the effectiveness of this drug in treating intractable limb pain from PDN resulting from both type 1 and 2 diabetes (Table 1) [14–18, 34]. A review of seven randomized controlled trials (RCTs) comparing pregabalin with placebo showed marginal benefits over placebo in decreasing average pain scores: 1.47 cm (placebo), 1.98 cm (150 mg pregabalin), 2.44 cm (300 mg pregabalin), and 2.75 cm (600 mg pregabalin) [35]. The mean follow-up was 4 to 12 weeks. Responder rates, representing the percentage of subjects with at least 50% improvement from baseline, varied from 40% to 49%, and placebo responder rates ranged from 14.5% to 23.0%. Adverse events (AEs) reported include dizziness, peripheral edema, somnolence, infection, and weight gain. Approximately 77% of patients prescribed pregabalin for PDN will discontinue the treatment within 1 year because of intolerable side effects or lack of efficacy [13]. In addition, the Neuropathic Pain Special Interest Group (NeuPSIG) of the International Association for the Study of Pain, which recently conducted a meta-analysis of 25 RCTs with pregabalin treatment of neuropathic pain, calculated that the number needed to treat (NNT) to achieve 50% pain reduction was 7.7 (range of 3.3–45.3); however, the safety profile for this medication is poor as the number needed to harm (NNH) is 13.9 [36]. Another commonly used class of medication for neuropathic pain, including PDN, consists of the serotonin-noradrenaline reuptake inhibitors (SNRIs). In an analysis of 10 RCTs for SNRIs, including seven duloxetine studies, the NeuPSIG reported a combined NNT of 6.4 (range of 4.2–30.2) and NNH of 11.8.

**Table 1:** Prior randomized controlled trial data for painful diabetic neuropathy

Treatment	Number	Average baseline pain	Average pain post-treatment	Responder rate	Length of follow-up	Reference
Pregabalin	76	6.5	4.0	40%	8 wks	[14]
	82	6.2	3.6	48%	5 wks	[15]
	82	6.7	4.3	39%	6 wks	[16]
	82	6.3	3.5	49%	13 wks	[17]
	101	6.7	3.7	46%	12 wks	[18]
	134	6.9	4.3	38%	12 wks	[19]
	66	6.5	4.8	21%	13 wks	[20]
	96	NR	NR	47%	16 wks	[21]
	123	7.1	2.0	NR	12 wks	[22]
Duloxetine	113	5.9	2.7	52%	12 wks	[23]
	116	5.5	3.0	50%	12 wks	[24]
	112	6.2	3.4	53%	12 wks	[25]
	106	5.5	2.8	57%	12 wks	[26]
	32	NR	NR	59%	8 wks	[27]
	138	7.1	4.0	42%	12 wks	[19]
	57	6.6	3.8	NR	8 wks	[28]
	67	NR	NR	28%	16 wks	[21]
Tapentadol	199	NR	NR	38%	15 wks	[29]
	12	6.5	3.9	NR	4 wks	[30]
	168	7.3	4.4	40%	15 wks	[31]
Low-frequency SCS	40	7.3	3.1	60%	6 mos	[32]
	22	7.3	4.0	47%	24 mos	[33]

Prior randomized controlled trial data for treatment of painful diabetic neuropathy with reported average pain scores (visual analog scale or numerical rating scale) and responder rates (reduction in pain of at least 50%). *Abbreviations*: *mos* months, *NR* not reported, *SCS* spinal cord stimulation, *wks* weeks

Low-frequency, paresthesia-based spinal cord stimulation (SCS) has also been shown to be effective in treating intractable pain associated with many peripheral neuropathies, including RCTs on PDN (Table 1) [32, 33, 37–42]. In a single-center, observational study, Pluijms et al. [39] reported that the median pain score of subjects treated with SCS decreased from 6 cm at baseline to 1.8 cm at 3 months on the visual analog scale (VAS) (range of 0–10 cm). However, at 12 months, the median pain score increased to 2.9 cm, and slightly over half the subjects (8/15 or 53%) were still responding to the therapy with at least 50% improvement in pain. In another study comparing SCS with best medical treatment, pain scores measured with the numerical rating scale (NRS) (range of 0–10) decreased from 7.3 and 6.7 (day and night, respectively) at baseline to 4 and 3.5 at 24 months [33]. Responder rates (subjects with at least 50% pain reduction) ranged from 47% (8/17, day) to 35% (6/17, night). Changes in pain scores in these studies were deemed to be both clinically and statistically significant.

Unlike traditional low-frequency, paresthesia-based SCS that seeks to induce paresthesias in the affected pain distribution, 10-kHz SCS therapy delivers paresthesia-independent, high-frequency stimulation by use of a unique waveform and uniform pulse width [43]. The therapy has demonstrated safety and superior effectiveness for the treatment of back and leg pain [44–49] and improved health-related quality of life [50]; 10-kHz SCS therapy has also been studied for the treatment of neuropathic limb pain, upper limb and neck pain, and pelvic pain ([51–54] Burgher A, Kosek P, Surrett S, Rosen S, Bromberg T, Gulve A, Kansal A, Wu P, McRoberts WP, Udeshi A, et al, 10 kHz SCS for treatment of chronic pain of the upper extremities: A post-market observational study, submitted). In a prospective multicenter study treating chronic intractable pain of the limbs from peripheral polyneuropathy using 10-kHz SCS therapy, subjects reported a decrease in mean pain score from 7.9 cm (± 0.3 standard error of the mean [SEM]) at baseline (*N* = 26) to 2.4 cm (± 0.5 SEM) at 6 months post-implant (*N* = 18), and 78% of subjects were deemed responders [55].

The current treatments for neuropathic pain secondary to PDN are suboptimal and there are substantial unmet needs [56]. In the proposed study, 10-kHz SCS therapy plus conventional medical management (CMM) will be compared with CMM alone for safety, clinical effectiveness, and cost-effectiveness in treating subjects diagnosed with chronic, neuropathic limb pain resulting from diabetic neuropathy. Subjects are allowed to cross to the alternative treatment arm after 6 months if they meet specific criteria. This protocol represents a pragmatic study designed to address current evidence gaps and meet treatment guidelines for the American Diabetes Association and the American Academy of Neurology.

## Methods/design

This is a multicenter, prospective, randomized controlled clinical study to document the comparative safety, clinical effectiveness, and cost-effectiveness of the addition of 10-kHz SCS therapy to CMM compared with CMM alone in subjects with chronic, intractable, neuropathic lower limb pain due to diabetic neuropathy. Enrollment of subjects will occur at multiple clinical sites only after institutional review board (IRB) approval and written informed consent from subjects have been obtained. Central ethical approval was provided by Western IRB (approval #1176998 received July 20, 2017), and local IRB approvals were obtained prior to recruiting at the corresponding sites. Subjects will be selected to participate in the study on the basis of the protocol’s inclusion (Table 2) and exclusion (Table 3) criteria. A panel of physician medical monitors independent of both the study sponsor and clinical investigator teams will review each consented subject to provide oversight of appropriate patient selection prior to randomization. Investigators will conduct the study in accordance with Good Clinical Practices as outlined in the US Code of Federal Regulations, the Declaration of Helsinki (version 2013), and other applicable regulatory requirements.

**Table 2:** Inclusion criteria

To participate in the study, subjects must meet all of the following inclusion criteria:	
1. Have been clinically diagnosed with diabetes, in accordance with the American Diabetes Association guidelines, as well as painful diabetic neuropathy of the lower limbs, and	
a. are symptomatic despite conservative therapy for a minimum of 12 months	
b. have tried pregabalin (Lyrica®) OR gabapentin (Neurontin®, Gralise®, etc.) administered at an adequate dose and for an appropriate duration in the investigator’s judgement	
c. have tried at least one other class of analgesic medication in addition to pregabalin/gabapentin	
d. are on a stable dosage of analgesic medications for at least 30 days	
2. Average pain intensity of at least 5 out of 10 cm on the visual analog scale in the lower extremities at enrollment.	
3. Have stable neurological status measured by motor, sensory, and reflex function as determined by the investigator.	
4. Be on a stable analgesic regimen, as determined by the investigator, for at least 30 days prior to assessing pain intensity as described in inclusion criterion #2 and be willing to stay on those medications with no dose adjustments until activation of the permanently implanted spinal cord stimulation (SCS) device (10-kHz SCS therapy group) or baseline assessment (conventional medical management–only group).	
5. Be 22 years of age or older at the time of enrollment.	
6. Be an appropriate candidate for the surgical procedures required in this study on the basis of the clinical judgment of the implanting physician.	
7. Be capable of subjective evaluation; able to read and understand English-written questionnaires; and able to read, understand, and sign the written informed consent in English.	
8. Be willing and able to give informed consent.	
9. Be willing and able to comply with study-related requirements, procedures, and scheduled visits.	
10. Have adequate cognitive ability to use a patient programmer and recharger as determined by the investigator.

**Table 3:** Exclusion criteria

To participate in the study, subjects must *not* meet any of the following exclusion criteria:	
1. Have a diagnosis of a lower limb mononeuropathy (e.g., causalgia and tibial or peroneal neuropathies), have had a lower limb amputation other than toes because of diabetes, or have large (≥3 cm) or gangrenous ulcers (or both) of the lower limbs.	
2. Have an average pain intensity of at least 3 out of 10 cm on the visual analog scale in the upper extremities because of diabetic neuropathy at enrollment.	
3. Currently have a hemoglobin A1c (HbA_1c_) of more than 10%.	
4. Have a body mass index of more than 45.	
5. Currently prescribed a daily opioid dosage of more than 120 mg morphine equivalents.	
6. Have a medical condition or pain in other area(s), not intended to be treated in this study, that could interfere with study procedures, accurate pain reporting, and/or confound evaluation of study endpoints, as determined by the investigator (such as primary headache, fibromyalgia, post-herpetic neuralgia, osteoarthritis, peripheral vascular disease, or small vessel disease).	
7. Have a current diagnosis of a progressive neurological disease such as multiple sclerosis, chronic inflammatory demyelinating polyneuropathy, rapidly progressive arachnoiditis, brain or spinal cord tumor, central deafferentation syndrome, complex regional pain syndrome, acute herniating disc, severe spinal stenosis, and brachial plexus injury, as determined by the investigator.	
8. Have a current diagnosis or condition such as a coagulation disorder, bleeding diathesis, platelet dysfunction, low platelet count, severely diminished functional capacity due to underlying cardiac/pulmonary disease, symptomatic uncontrolled hypertension, progressive peripheral vascular disease, or uncontrolled diabetes mellitus that presents excess risk for performing the procedure, as determined clinically by the investigator.	
9. Have experience with spinal cord stimulation, dorsal root ganglion stimulation, peripheral nerve field stimulation, or peripheral nerve stimulation for chronic intractable pain.	
10. Have significant spinal stenosis, objective evidence of epidural scarring and/or any signs or symptoms of myelopathy as determined by the investigator on the basis of magnetic resonance imaging (MRI) conducted within the past 12 months.	
11. Any history of surgery on the posterior elements (laminectomy, posterior fusion), resulting in a compromised epidural space, as determined by the investigator.	
12. Be benefitting from an interventional procedure or surgery (or both) to treat lower limb pain. (Subjects should be enrolled at least 30 days from last benefit.)	
13. Have an existing drug pump or another active implantable device such as a pacemaker or both.	
14. Have a condition currently requiring or likely to require the use of diathermy or MRI that is inconsistent with Senza system guidelines in the Physician’s Manual.	
15. Have either a metastatic malignant neoplasm or untreated local malignant neoplasm.	
16. Have a life expectancy of less than 1 year.	
17. Have a local infection at the anticipated surgical entry site or an active systemic infection.	
18. Be pregnant or plan to become pregnant during the study. Women of childbearing potential who are sexually active must use a reliable form of birth control, be surgically sterile, or be at least 2 years post-menopausal.	
19. Have within 6 months of enrollment a significant untreated addiction to dependency-producing medications, alcohol, or illicit drugs.	
20. Be concomitantly participating in another clinical study.	
21. Be involved in an injury claim under current litigation.	
22. Be a recipient of temporary Social Security Disability Insurance benefits because of chronic pain.	
23. Have a pending or approved worker’s compensation claim.	
24. Have evidence of an active disruptive psychological or psychiatric disorder or other known condition significant enough to impact perception of pain, compliance with intervention and/or ability to evaluate treatment outcome, as determined by a psychologist in the last 12 months.	

### Randomization

Subjects meeting the requirements for inclusion and exclusion criteria will be randomly assigned 1:1 to 10-kHz SCS therapy delivered by a Senza SCS System (Nevro Corp., Redwood City, CA, USA) plus CMM or to CMM alone. The randomization for each site will be performed by a block randomization method developed by an independent statistician. Randomization will be stratified by average baseline pain VAS score and the baseline hemoglobin A1c (HbA_1c_) level. Thus, there will be four strata per site. Concealed allocation will be achieved via computer assignment of the treatment arm, and investigational site staff and study sponsor personnel will be unaware of the block size and randomization list. Owing to the nature of the treatments, specifically an implanted medical device compared with CMM, blinding subjects or investigator teams to the treatment assignment is not feasible. Subjects randomly assigned to either treatment group will have the potential to cross over to the alternative treatment arm at the 6-month visit if they meet all of the following criteria: (1) less than 50% lower limb pain relief from baseline, (2) documented subject dissatisfaction with the treatment (“dissatisfied” or “very dissatisfied” on subject satisfaction measure), and (3) investigator agreement with crossover.

### Sample size

Up to 432 subjects will be screened at multiple clinical sites in the US to obtain a total of 216 randomly assigned subjects, resulting in approximately 108 subjects assigned to each treatment group. Subjects will continue with their respective treatments through the 3-month primary endpoint with an expected 10% attrition rate, resulting in approximately 97 subjects in each group at the primary endpoint. This is the sample size required on the basis of the following assumptions: a 60% responder rate for the 10-kHz SCS therapy group (80% trial success rate and 75% responders at 3 months among permanent implant subjects), a 36% responder rate for the CMM-only group, 90% power, and two-sided type I error of 0.05. Interim analysis will be performed to reassess sample size assumptions when 25% of the subjects reach the 3-month primary endpoint.

### Outcomes assessed

**Fig. 1:**
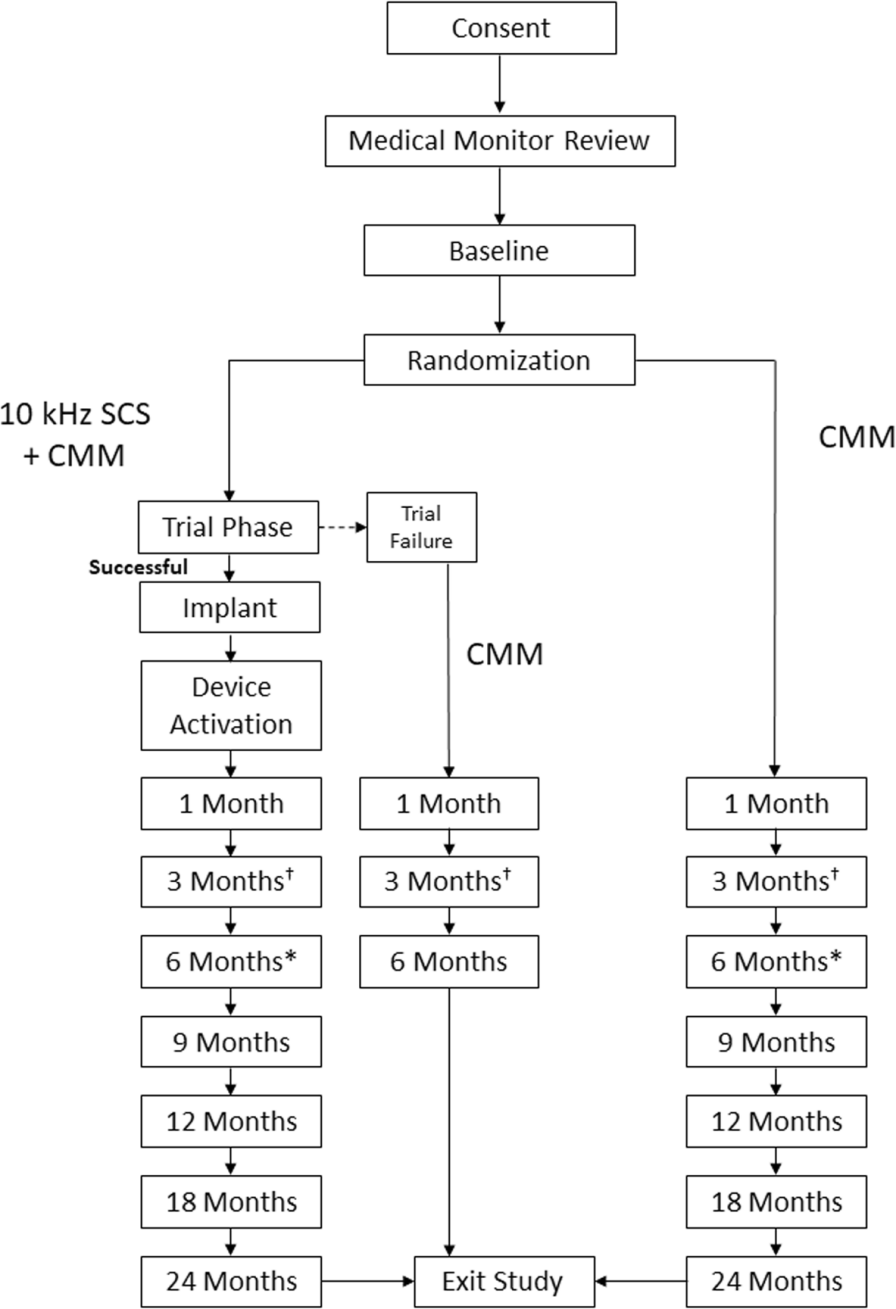
Summary of the sequence of study-related assessments, procedures, and activities

Outcome data will be collected at baseline, trial and implant (10-kHz SCS therapy group), and defined follow-up intervals (Fig. 1, Table 4). Data collection will include measures of pain, health-related quality of life, function, subject satisfaction, health-care utilization, and medication usage, including treatments for pain relief and diabetic management (Table 5). Data will be collected by using electronic case report forms (eCRFs) via an Electronic Data Capture (EDC) system (M-Core, Medrio Inc., San Francisco, CA, USA). Data will be collected by the site research staff and entered directly into eCRFs in the EDC system at the investigational sites. The clinical site will record data on outcome variables as well as AEs should they occur. Subject confidentiality will be maintained, and each subject will be identified by his or her subject number.

**Table 4:** Participant data collection time line

Assessment	Enrollment			Trial and permanent phase (SCS only)				Follow-up phase						
Visit	Consent	Entry criteria	Baseline assessment	Trial implant	End of trial	Permanent implant	Device activation	1-month visit	3-month visit	6-month visit	9-month visit	12-month visit	18-month visit	24-month visit
Informed consent	X													
Medication usage		X	X	X	X	X	X	X	X	X	X	X	X	X
Health-care utilization			X					X	X	X	X	X	X	X
Pain assessment (VAS)		X	X		X			X	X	X	X	X	X	X
Weight		X	X						X	X		X		X
Neuropathic pain assessment (DN4)			X		X			X	X	X		X		X
Modified Neuropathy Symptom Score (NSS)			X		X			X	X	X		X		X
Brief Pain Inventory (BPI-DPN)			X					X	X	X	X	X	X	X
Pain experience (SF-MPQ-2)			X						X	X		X		X
Diabetes quality of Life (DQOL)			X						X	X		X		X
Quality of life assessment (EQ-5D-5L)			X						X	X		X		X
Pain and sleep assessment (PSQ-3)			X					X	X	X	X	X	X	X
Patient Global Impression of Change (PGIC)									X	X		X		X
Clinical Global Impression of Change (CGIC)									X	X		X		X
Global Assessment of functioning (GAF)			X					X	X	X	X	X	X	X
Six-minute walk test (6MWT)			X						X			X		X
Hemoglobin A1c		X							X	X		X	X	X
Subject satisfaction									X	X		X		X
Neurological assessment			X		X				X	X		X		X
Work status and disability			X							X		X		X
Adverse event monitoring		X	X	X	X	X	X	X	X	X	X	X	X	X
Wound assessment			X				X	X	X	X	X	X	X	X

*Abbreviations*: *SCS* spinal cord stimulation, *VAS* visual analog scale

**Table 5:** Outcomes

Outcome	Variables	Reference
Pain		
Pain assessment (VAS)	0–10 cm	[57]
Responder rates	Percentage of subjects with ≥50% pain relief compared to baseline	NA
Remitter rates	Percentage of subjects with ≤3.0 cm pain VAS for at least 6 months	[58]
Douleur Neuropathique 4 (DN4)	0–10	[59]
Modified Neuropathy Symptom Score (NSS)	0–9	[60]
Brief Pain Inventory for Diabetic Peripheral Neuropathy (BPI-DPN)	Pain severity: 0–10	[61]
	Pain-related interference: 0–10	
Short-Form McGill Pain Questionnaire (SF-MPQ-2)	Continuous pain: 0–10	[62]
	Intermittent pain: 0–10	
	Neuropathic pain: 0–10	
	Affective descriptors: 0–10	
	Total: 0–10	
Health-related quality of life		
Diabetes Quality of Life (DQOL)	Impact: 1–5	[63]
	Satisfaction: 1–5	
	Diabetes worry: 1–5	
	Social worry: 1–5	
	Total: 1–5	
EuroQol Five Dimensions Questionnaire (EQ-5D-5L)	Index value (US): −0.109-1.0	[64]
	Health VAS: 0–100	
Global Assessment of Functioning (GAF)	0–100	DSM-IV
Patient Global Impression of Change (PGIC) and Clinical Global Impression of Change (CGIC)	No change, almost the same, a little bit better, somewhat better, moderately better, or a great deal better	NA
Sleep (PSQ-3)	0–10 cm	[65]
Subject satisfaction	Very dissatisfied, dissatisfied, not sure, satisfied, or very satisfied	NA
Medications – Analgesics and Diabetic Control		
Medication usage	Increased, no change, decreased, eliminated	NA
Dosage	Average dose over time	NA
Function and Health		
Six-minute walk test	Total meters walked	[66]
	Shortness of breath: 0–10	
	Fatigue: 0–10	
Body mass index	Underweight: <18.5 kg/m^2^	CDC
	Normal: 18.5–24.9 kg/m^2^	
	Overweight: 25.0–29.9 kg/m^2^; obese: >30.0 kg/m^2^	
Hemoglobin A1c (HbA_1c_)	Normal: 4.0%–5.6%	ADA
	Prediabetes: 5.7%–6.4%	
	Diabetes: ≥6.5%	
Wound healing, including ulcer surveillance	Change in greatest diameter over time	NA
Health-care utilization	Type, reason, and tests/treatments received during physician, emergency department, and hospital visits	NA
Work status	Current employment and reason for not working, if applicable	NA
Safety		
Neurological assessments: lower limb motor function, L1–S1 sensation to light touch, pinprick and Semmes–Weinstein 10-g monofilament sensory testing of the feet, patellar and Achilles reflexes, Babinski response	Clinically meaningful deficit, no change, or clinically meaningful improvement in motor, sensory, and reflexes, compared with baseline	NA
Adverse events	Grade I-IV	NA

*Abbreviations*: *ADA* American Diabetes Association, *CDC* Centers for Disease Control and Prevention, *DSM-IV* Diagnostic and Statistical Manual of Mental Disorders - 4th edition, *NA* not applicable, *VAS* visual analog scale

### Statistical analysis

The primary endpoint of this study is a composite of safety and effectiveness at 3 months, specifically the percentage of subjects who respond to treatment without a clinically meaningful neurological deficit compared with baseline. A responder is defined as a subject with at least 50% reduction in lower limb pain from baseline. For each subject and all analyses, the right and left lower limb VAS scores collected during a single visit will be averaged together to generate a lower limb pain score. In addition to the primary endpoint, several secondary and tertiary endpoints will be evaluated (Tables 6 and 7).

**Table 6:** Secondary study endpoints

1. Difference between the treatment groups in proportion of subjects with a lower limb pain visual analog scale (VAS) score of not more than 3.0 cm at 3 months.	
2. Difference between the treatment groups in crossover rates.	
3. Difference between the treatment groups in responder rates at 6 months.	
4. Difference between the treatment groups in the proportion of remitters (remission is defined as having a lower limb pain VAS score of not more than 3.0 cm for at least 6 months) at 6 months.	
5. Difference between the treatment groups in the proportion of subjects with improvement from baseline in neurological assessment (motor, sensory, or reflex) at 3 months.	
6. Difference between the treatment groups in the proportion of subjects with overall improvement from baseline in neurological assessment (motor, sensory, or reflex) at 6 months.	
7. Difference between the treatment groups in changes in health-related quality of life as assessed by the EuroQol Five Dimensions questionnaire (EQ-5D-5L) at 6 months.	
8. Difference between the treatment groups in the average percentage change from baseline in hemoglobin A1c (HbA_1c_) levels at 6 months.

**Table 7:** Tertiary study endpoints

• Difference between the treatment groups in the average percentage change from baseline in lower limb pain visual analog scale (VAS) scores at 3 and 6 months. Within-group evaluations will be carried out at 12 and 24 months.	
• Difference between the treatment groups in proportion of subjects with at least 30% improvement in lower limb pain VAS at 3 and 6 months. Within-group evaluations will be carried out at 12 and 24 months.	
• Within-group evaluation of proportion of remitters at 12 and 24 months.	
• Within-group evaluation of responder rates at 12 and 24 months.	
• Within-group evaluation of proportion of subjects with improvement from baseline in neurological assessment (motor, sensory, or reflex) at 12 and 24 months.	
• Difference between the treatment groups in numbers needed to treat (NNTs) based on responder rates at 3 and 6 months. Within-group evaluations will be carried out at 12 and 24 months.	
• Difference between the treatment groups in average percentage change from baseline in opioid dosage at 3 and 6 months. Within-group evaluations will be carried out at 12 and 24 months.	
• Difference between the treatment groups in average percentage change from baseline in painful diabetic neuropathy–specific analgesic dosages at 3 and 6 months. Within-group evaluations will be carried out at 12 and 24 months.	
• Difference between the treatment groups in average percentage change from baseline in hemoglobin A1c (HbA_1c_) levels at 3 months. Within-group evaluation will be carried out at 12 and 24 months.	
• Difference between the treatment groups in average percentage change from baseline in diabetic control medication dosages at 3 and 6 months. Within-group evaluations will be carried out at 12 and 24 months.	
• Difference between the treatment groups in average percentage change from baseline in body mass index at 3 and 6 months. Within-group evaluations will be carried out at 12 and 24 months.	
• Difference between the treatment groups in the average percentage change from baseline on distance covered during the 6-minute walk test (6MWT) at 3 months. Within-group evaluations will be carried out at 12 and 24 months.	
• Difference between the treatment groups in the change over time in size of lower limb wounds at 3 and 6 months. Within-group evaluations will be carried out at 12 and 24 months.	
• Difference between the treatment groups at 3 and 6 months in health economic outcomes, including (1) health-care utilization (i.e., medications, office visits, emergency room visits, hospital admissions, medical tests, etc.), (2) employment status, and (3) health-related quality of life as assessed by the EuroQol Five Dimensions questionnaire (EQ-5D-5L) and the Diabetes Quality of Life (DQOL) measure. Within-group evaluations will be carried out at 12 and 24 months.	

Descriptive statistics will be used to summarize all subject baseline and outcome data collected during the study. Continuous variables will be summarized by using means, standard deviations, medians, and ranges. Categorical variables will be summarized in frequency distributions. Statistical tests appropriate to the endpoint being examined will be used and identified. Parametric tests (e.g., Student’s *t* tests) will be used if the distributional properties of the data are suitable. If parametric tests are not indicated, the associated non-parametric tests (e.g., Mann–Whitney tests and Fisher’s exact tests) will be used. A two-sided *P* value of 0.05 or less for the primary endpoint will be considered evidence of statistical significance. Reported *P* values for all other tests will be considered nominal and unadjusted for multiple testing but without conclusions regarding statistical significance levels.

Analysis populations defined for the study include intention-to-treat (ITT) and per protocol (PP). The ITT population includes all subjects randomly assigned to the CMM and CMM plus 10-kHz SCS study groups. This is considered the safety population for purposes of reporting on any reported AEs. The PP population includes all ITT subjects who complete the 3-month primary assessment. The primary analysis population for the primary study endpoint is the ITT population. Secondary analyses will be performed in the PP population. The responder rates will be compared between groups with a Fisher’s exact test. Hierarchical testing will be carried out on the secondary endpoints listed in Table 6. Additional analyses will be performed for subjects who cross over to 10-kHz SCS treatment by using their data collected during the initial 6 months of CMM treatment as a control. Health economic outcomes will be assessed from health-care utilization, medication, work status, and health-related quality of life data.

### Safety

A clinical events committee (CEC) will be convened to provide oversight during the study. This expert panel will serve in an advisory role to review safety data at interim points during the study, including the review of AEs and adjudication of the relatedness and seriousness of serious AEs. If needed, the CEC will also review unanticipated serious adverse device events on an urgent basis. The CEC will consist of clinicians with expertise in pain management, neurology, and endocrinology. Data review meetings will be held at regular intervals with the option for an ad hoc meeting at any time if an imminent subject safety issue arises. The CEC will have one physician member representing the study sponsor and the other physician members will be independent of the study sponsor and clinical investigators.

## Discussion

In the treatment of neuropathic pain secondary to PDN, 10-kHz SCS has the potential to deliver safe and effective pain relief that is non-pharmacologic and paresthesia-independent. This would be an important development in the field because currently available treatment options for this condition, including pharmacologic agents and conventional SCS, are not adequate for all patients [56].

Pharmacologic options for treating neuropathic pain include anticonvulsants such as pregabalin, which has shown clinical efficacy in treating neuropathic pain due to PDN in high-quality studies [14–18, 34]. These trials were placebo-controlled and had large sample sizes but only short-term follow-up (lasting from 4 to 13 weeks). A study that pooled data from seven pregabalin trials to improve statistical power found that pain reductions were modest, the average NRS reduction was 2.75 for pregabalin (600 mg/day), and a majority of patients (53%) did not respond to the drug, defined as pain relief of at least 50% [35]. Duloxetine has also demonstrated pain relief for PDN in several well-designed RCTs. With follow-up lasting 8 to 16 weeks, reported responder rates ranged from 28% to 59% [19, 21, 23–28].

Conventional, low-frequency SCS has also been tested for treating neuropathic pain in patients with PDN. Median patient-reported NRS scores were reduced by an average of 3.1 after 12 months in a small study of 15 patients [39] and 3.3 after 24 months of stimulation in a study of 22 patients [33]. Although the long-term durability of these results is promising, the magnitude of pain relief was modest, similar to that offered by medication, and about half of the subjects did not respond to SCS treatment in each study. These results, combined with the technical difficulties presented by targeting paresthesia-dependent stimulation to the feet, support the development of additional options for treating this patient population.

The study described here will help determine the efficacy of 10-kHz SCS in patients with neuropathic pain due to PDN, a currently underserved patient population. About 100 patients will be randomly assigned to each treatment arm recruited at multiple sites throughout the US, which will result in a greater power to detect statistically and clinically meaningful results compared with prior SCS studies. Follow-up will continue for 24 months to demonstrate long-term outcomes compared with prior pharmacological data. Study sites include both large academic centers and independent pain clinics in geographically diverse areas that will provide a representative patient population. A limitation of the study is the contribution of employees of the sponsor to protocol design and data analysis as this has the potential to introduce bias. Multiple measures have been taken to minimize bias: the participation of outside medical experts in the design of the study; recruitment of independent physician investigators who are responsible for patient selection, data collection, and oversight of study conduct at their sites; concealed allocation of treatment; and the involvement of the CEC to monitor study safety. The primary endpoint results will be reported for the ITT population, and secondary and tertiary outcomes will be reported for the PP population.

Another potential limitation is the cost of the proposed treatment in this study versus CMM. SCS is typically an outpatient procedure during which percutaneous leads and battery are fully implanted. The device in this study is rechargeable, and the expected battery life is in excess of 10 years when used at typical therapeutic settings. This study will collect data on health-care utilization, health-related quality of life, and medication usage to address whether the upfront cost for an implanted medical device can be justified by benefit over the life of the product, similar to what has been reported previously for SCS [67–69] (Additional file 1).

## Conclusions

The SENZA-PDN study will be the largest RCT conducted to date using SCS in subjects with PDN. This prospective multicenter study will determine whether 10-kHz SCS improves clinical outcomes and health-related quality of life and is a cost-effective treatment for PDN. The current treatments for neuropathic pain secondary to PDN are suboptimal and have limited effectiveness and intolerable side effects. Primary endpoint data are expected in 2020, and 24-month data in 2022.

## Trial status

Protocol CA2016–5 US SENZA-PDN-1, revision D (March 19, 2019). The first subject was randomly assigned in October 2017 and the last in August 2019.

## Supplementary information

**Additional file 1.** SPIRIT (Standard Protocol Items: Recommendations for Interventional Trials) 2013 Checklist.

## Data Availability

Not applicable.

## Abbreviations

AE: Adverse event
CEC: Clinical events committee
CMM: Conventional medical management
eCRF: Electronic case report forms
EDC: Electronic Data Capture
IRB: Institutional review board
ITT: Intention-to-treat
NeuPSIG: Neuropathic Pain Special Interest Group
NNH: Number needed to harm
NNT: Numbers needed to treat
NRS: Numerical rating scale
PDN: Painful diabetic neuropathy
PP: Per protocol
RCT: Randomized controlled trial
SEM: Standard error of the mean
SNRI: Serotonin-noradrenaline reuptake inhibitor
VAS: Visual analog scale
