# Supplementary material for: High-frequency spinal cord stimulation at 10 kHz for the treatment of painful diabetic neuropathy: design of a multicenter, randomized controlled trial (SENZA-PDN)
Source: Trials. 2020 Jan 15;21:87. doi: 10.1186/s13063-019-4007-y (PMC6961392; doi:10.1186/s13063-019-4007-y)
Supplement: Supplementary file 1 — Additional file 1. SPIRIT (Standard Protocol Items: Recommendations for Interventional Trials) 2013 Checklist. [file 13063_2019_4007_MOESM1_ESM.docx]

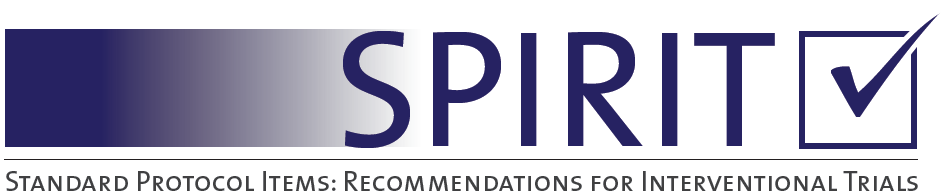


SPIRIT 2013 Checklist: Recommended items to address in a clinical trial protocol and related documents*

| Section/item | ItemNo | Description |
| --- | --- | --- |
| **Administrative information** | | |
| Title | 1  P 1 | High Frequency Spinal Cord Stimulation at 10 kHz for the Treatment of Painful Diabetic Neuropathy: Design of a Multicenter, Randomized, Controlled Trial (SENZA-PDN) |
| Trial registration | 2a  P 2 | ClincalTrials.gov - NCT03228420 (registered July 24, 2017) |
|  | 2b  P 2 | <https://clinicaltrials.gov/ct2/show/NCT03228420?term=nevro&rank=6> |
| Protocol version | 3  P 10 | Protocol # CA2016-5 US SENZA-PDN-1, Revision D (March 19, 2019)  Revision chronology:   - 26May2017 original (Revision A) - 14Mar2018 Revision B   - Protocol enhancements and clarifications - 28Mar2018 Revision C   - Protocol clarifications - 19Mar2019 Revision D   - Protocol clarifications and revision of enrolment estimate |
| Funding | 4  P 11 | Funded by Nevro Corp. |
| Roles and responsibilities | 5a  P 1 | Nagy A. Mekhail, MD PhD  Department of Pain Management  Cleveland Clinic  Cleveland, OH, USA  Charles E. Argoff, MD  Department of Neurology  Albany Medical College  Albany, NY, USA  Rod S. Taylor, MSc PhD  Institute of Health and Well Being  University of Glasgow  Glasgow, Scotland, UK  College of Medicine and Health  University of Exeter  Exeter, England, UK |
|  |  | Christian Nasr, MD  Department of Endocrinology  Cleveland Clinic  Cleveland, OH, USA  David L. Caraway, MD PhD  Nevro Corp.  Redwood City, CA, USA  Bradford E. Gliner, MS  Nevro Corp.  Redwood City, CA, USA  Jeyakumar Subbaroyan, PhD  Nevro Corp.  Redwood City, CA, USA  Elizabeth S. Brooks, PhD  Nevro Corp.  Redwood City, CA, USA  All authors contributed to the design of the study protocol and approved of the final manuscript. |
|  | 5b  NA | Brad Gliner, MS  Vice President, Clinical and Regulatory Affairs  Nevro Corp.  (650) 433-3228  gliner@nevro.com |
|  | 5c  NA | The sponsor has a role in study design, management, data analysis and interpretation, as well as writing reports and deciding on publication of study-wide results.  Investigators collect the data and will be involved in analysis, interpretation, and composing publications. Investigators may publish individual site data after the main study publication. |
|  | 5d  P 6 | Steering Committee – responsible for development of the protocol and providing guidance throughout the study. Members include individuals from sponsor as well as independent from sponsor.  Medical Monitors – independent physicians who review medical records for all subjects prior to randomization to assess suitability for the study. |

| Introduction |  |  |
| --- | --- | --- |
| Background and rationale | 6a  P 3-6 | Globally, 422 million people have diabetes, resulting in US$1.7 trillion in direct and indirect costs. (1) Data from the Centers for Disease Control and Prevention (CDC) estimate there are currently 29 million people in the United States living with diabetes, and another 86 million with prediabetes, resulting in $245 billion in healthcare costs and lost productivity. (2) Approximately 20% of patients with diabetes will develop painful diabetic neuropathy (PDN), (3) a debilitating, progressive chronic pain condition that significantly impacts their quality of life.  Peripheral neuropathy from damage to peripheral nerves may result in pain, numbness, and/or weakness in the affected limb. Damage may affect small (myelinated Aδ and unmyelinated C) fibers along with injury to large myelinated fibers. One of the classifications for peripheral neuropathy is based on whether the damage is to a single nerve (mononeuropathy) or multiple nerves (polyneuropathy). The causes of polyneuropathy may include metabolic (e.g., chronic renal failure), endocrine disorders (e.g., painful diabetic neuropathy or PDN), treatment induced toxicity (e.g., radiation, chemotherapy, or alcohol-induced neuropathy), infection (post-herpetic neuralgia caused by Herpes Zoster virus, Lyme disease), autoimmune disorders (Guillain-Barre’ syndrome, Charcot-Marie-Tooth neuropathy), compression (carpal tunnel syndrome, tarsal tunnel syndrome, ulnar neuropathy, peroneal neuropathy) and trauma (trauma-induced neuropathy). Nearly half of peripheral neuropathy patients are diagnosed as idiopathic. (4)  The American Chronic Pain Association estimates that more than 15 million people in the U.S. and Europe have some degree of neuropathic pain. More than 2 in 100 persons are estimated to have peripheral neuropathy; the incidence rises to 8 in 100 for people aged 55 or older. (5) In Europe, the prevalence of PDN ranged from 5.8 to 34.0%. (6) The incidence of PDN was reported to be 0.72 per 1000 persons per year for the Netherlands, (7) and 0.64–0.69 per 1000 persons per year in the UK. (8) PDN is very taxing to the individual patient’s life due to suffering, impaired quality of life, and increased disability, (9; 10) as well as to society as a whole due to the significant impact on the workforce and the increased cost of healthcare. (11; 12) |
|  |  | Anticonvulsant medications, including gabapentin and pregabalin, are among the most commonly prescribed medications for neuropathic pain due to PDN. (13) Pregabalin, or (*S*)-3-(aminomethyl)-5-methylhexanoic acid, is an analog of the inhibitory neurotransmitter gamma-aminobutyric acid (GABA). It is a compound that acts on the central nervous system producing analgesic, anticonvulsant, and anxiolytic effects. Clinical studies have demonstrated the effectiveness of this drug in treating intractable limb pain from PDN resulting from both type 1 and 2 diabetes (Table 1). (14-19) A review of 7 randomized controlled trials comparing pregabalin with placebo showed marginal benefits over placebo in decreasing average pain scores: 1.47 cm (placebo), 1.98 cm (150 mg pregabalin), 2.44 cm (300 mg pregabalin) and 2.75 cm (600 mg pregabalin). (20) Mean follow-up was 4-12 weeks. Responder rates, representing the percent of subjects with at least 50% improvement from baseline, varied from 40-49% while placebo responder rates ranged 14.5-23.0%. Adverse events reported include dizziness, peripheral edema, somnolence, infection and weight gain. Approximately 77% of patients prescribed pregabalin for PDN will discontinue the treatment within one year due to intolerable side effects or lack of efficacy. (13) In addition, a recent meta-analysis of RCTs with pregabalin treatment of neuropathic pain calculated the Numbers Needed to Treat (NNT) to achieve 50% pain reduction is 7.7; however, the safety profile for this medication is poor as the Number Needed to Harm (NNH) is 13.9. (21)  Low-frequency, paresthesia-based spinal cord stimulation (SCS) has also been shown to be effective in treating intractable pain associated with many peripheral neuropathies, including randomized, controlled trials on PDN (Table 1). (22-29) In a single center, observational study, Pluijms et al. (24) reported that the median pain score of subjects treated with SCS decreased from 6 cm at baseline to 1.8 cm at 3 months on the visual analog scale (VAS, range: 0-10 cm). However, at 12 months the median pain score increased up to 2.9 cm with slightly over half the subjects (8/15 or 53%) still responding to the therapy with at least 50% improvement in pain. In another study comparing SCS with best medical treatment, pain scores measured with the numerical rating scale (NRS, range: 0-10) decreased from 7.3 and 6.7 (day and night, respectively) at baseline to 4 and 3.5 at 24 months. (27) Responder rates (subjects with ≥ 50% pain reduction) ranged from 47% (8/17, day) to 35% (6/17, night). Changes in pain scores in these studies were deemed both clinically and statistically significant. |
|  |  | Unlike traditional low-frequency, paresthesia-based SCS that seeks to induce paresthesias in the affected pain distribution, 10 kHz SCS therapy delivers paresthesia-independent, high frequency stimulation, by use of a unique waveform and uniform pulse width. (30) The therapy has demonstrated safety and superior effectiveness for the treatment of back and leg pain (31-34) as well as improved health-related quality of life. (35) 10 kHz SCS therapy has also been studied for the treatment of neuropathic limb pain as well as pelvic pain. (36-38) In a prospective, multicenter study treating chronic intractable pain of the limbs from peripheral polyneuropathy using 10 kHz SCS therapy, subjects reported a decrease in mean pain score from 7.9 cm (±0.3, standard error of the mean [SEM]) at baseline (N=26) to 2.4 cm (±0.5, SEM) at 6 months post-implant (N=18), with 78% of subjects deemed responders. (39)  The current treatments for neuropathic pain secondary to PDN are suboptimal with substantial unmet needs. (40) In the proposed study, 10 kHz SCS therapy plus CMM will be compared with CMM alone for safety, clinical effectiveness, and cost-effectiveness in treating subjects diagnosed with chronic, neuropathic limb pain resulting from diabetic neuropathy. Subjects are allowed to cross to the alternative treatment arm after 6 months if they meet specific criteria. This protocol represents a pragmatic study designed to address current evidence gaps and meet treatment guidelines for the American Diabetes Association (ADA) and the American Academy of Neurology (AAN). |
|  | 6b  NA | Currently, there is not a single best medical treatment for PDN but a number of primarily pharmacological interventions applied sequentially and in combination. The choice of CMM as a comparator allows subjects to be optimized according to the current standard of care and is representative of the patient population as a whole. |
| Objectives | 7  P 2 | The purpose of this post-market study is to document comparative safety, clinical effectiveness, and cost-effectiveness of the addition of 10 kHz SCS to CMM compared with CMM alone in subjects with chronic, intractable, neuropathic lower limb pain due to diabetic neuropathy (Painful Diabetic Neuropathy or PDN). |
| Trial design | 8  P 2 | This study is a multi-center, prospective, randomized (1:1) comparison of the two treatments. |
| Methods: Participants, interventions, and outcomes | | |
| Study setting | 9  P 6 | Subjects will be recruited at multiple sites in the US, including academic centers and community clinics. |
| Eligibility criteria | 10  Tables 2 & 3 | Inclusion criteria:   1. Have been clinically diagnosed with diabetes, according to the American Diabetes Association guidelines, as well as painful diabetic neuropathy (PDN) of the lower limbs, and:   are symptomatic despite conservative therapy for a minimum of 12 months  have tried pregabalin (Lyrica^®^) OR gabapentin (Neurontin^®^, Gralise^®^, etc.) administered at an adequate dose and for an appropriate duration, in the Investigator’s judgement  have tried at least one other class of analgesic medication in addition to pregabalin/gabapentin  are on a stable dosage of analgesic medications for at least 30 days   1. Average pain intensity of ≥ 5 out of 10 cm on the VAS in the lower extremities at enrollment. 2. Have stable neurological status measured by motor, sensory and reflex function as determined by the investigator. 3. Be on a stable analgesic regimen, as determined by the Investigator, for at least 30 days prior to assessing pain intensity as described in inclusion criterion #2, and be willing to stay on those medications with no dose adjustments until activation of the permanently implanted SCS device (HF10 therapy group) or baseline assessment (CMM only group). 4. Be 22 years of age or older at the time of enrollment. 5. Be an appropriate candidate for the surgical procedures required in this study based on the clinical judgment of the implanting physician. 6. Be capable of subjective evaluation, able to read and understand English-written questionnaires, and able to read, understand and sign the written informed consent in English. 7. Be willing and capable of giving informed consent. 8. Be willing and able to comply with study-related requirements, procedures, and scheduled visits. 9. Have adequate cognitive ability to use a patient programmer and recharger as determined by the Investigator.   Exclusion criteria:   1. Have a diagnosis of a lower limb mononeuropathy (e.g., causalgia and tibial or peroneal neuropathies), have had a lower limb amputation other than toes due to diabetes, or have large (≥3 cm) and/or gangrenous ulcers of the lower limbs. 2. Have an average pain intensity of ≥ 3 out of 10 cm on the VAS in the upper extremities due to diabetic neuropathy at enrollment. |
|  |  | 1. Currently have a hemoglobin A1c (HbA1c) > 10%. 2. Have a BMI > 45. 3. Currently prescribed a daily opioid dosage > 120 mg morphine equivalents. 4. Have a medical condition or pain in other area(s), not intended to be treated in this study, that could interfere with study procedures, accurate pain reporting, and/or confound evaluation of study endpoints, as determined by the Investigator (such as primary headache, fibromyalgia, post-herpetic neuralgia, osteoarthritis, peripheral vascular disease, or small vessel disease). 5. Have a current diagnosis of a progressive neurological disease such a multiple sclerosis, chronic inflammatory demyelinating polyneuropathy, rapidly progressive arachnoiditis, brain or spinal cord tumor, central deafferentation syndrome, Complex Regional Pain Syndrome, acute herniating disc, severe spinal stenosis and brachial plexus injury, as determined by the Investigator. 6. Have a current diagnosis or condition such as a coagulation disorder, bleeding diathesis, platelet dysfunction, low platelet count, severely diminished functional capacity due to underlying cardiac/pulmonary disease, symptomatic uncontrolled hypertension, progressive peripheral vascular disease or uncontrolled diabetes mellitus that presents excess risk for performing the procedure, as determined clinically by the Investigator. 7. Have prior experience with SCS, dorsal root ganglion (DRG) stimulation, peripheral nerve field stimulation (PNfS), or peripheral nerve stimulation (PNS) for chronic intractable pain. 8. Have significant spinal stenosis, objective evidence of epidural scarring and/or any signs or symptoms of myelopathy as determined by the Investigator based on MRI conducted within the past 12 months. 9. Any previous history of surgery on the posterior elements (laminectomy, posterior fusion) resulting in a compromised epidural space, as determined by the Investigator. 10. Be benefitting from an interventional procedure and/or surgery to treat lower limb pain (Subjects should be enrolled at least 30 days from last benefit). 11. Have an existing drug pump and/or another active implantable device such as a pacemaker. 12. Have a condition currently requiring or likely to require the use of diathermy or MRI that is inconsistent with Senza system guidelines in the Physician’s Manual. 13. Have either a metastatic malignant neoplasm or untreated local malignant neoplasm. |
|  |  | 1. Have a life expectancy of less than one year. 2. Have a local infection at the anticipated surgical entry site or an active systemic infection. 3. Be pregnant or plan to become pregnant during the study. Women of childbearing potential who are sexually active must use a reliable form of birth control, be surgically sterile, or be at least 2 years post-menopausal. 4. Have within 6 months of enrollment a significant untreated addiction to dependency producing medications, alcohol or illicit drugs. 5. Be concomitantly participating in another clinical study. 6. Be involved in an injury claim under current litigation. 7. Be a recipient of temporary Social Security Disability Insurance (SSDI) benefits due to chronic pain. 8. Have a pending or approved worker’s compensation claim. 9. Have evidence of an active disruptive psychological or psychiatric disorder or other known condition significant enough to impact perception of pain, compliance with intervention and/or ability to evaluate treatment outcome, as determined by a psychologist in the last 12 months.   Investigators must have adequate experience with SCS procedures prior to participating in the study. |
| Interventions | 11a  P 6 | **10 kHz SCS (**Senza System PMA P130022) has been approved by the FDA with Indications for Use including the management of neuropathic pain of the limbs as described in this Investigational Plan. **Treatment of subjects with peripheral neuropathies that result in limb pain is therefore an on-label use of the Senza System. Typically, subjects will undergo temporary trial stimulation to assess effectiveness. If at least 50% pain relief is achieved compared to baseline the subject is eligible for implantation of a permanent 10 kHz stimulation system.**  **CMM will follow the Investigators’ standard of care and/or published clinical guidelines** (Dworkin, 2010)**. Treatments include, but are not limited to, pharmacological agents, physical therapy, cognitive therapy, chiropractic care, nerve blocks, and other non-invasive** or minimally invasive **therapies.** |
|  | 11b  NA | Subjects can opt to turn off or have an implanted device removed at any time. Changes in CMM treatments should follow standard medical practice. |
|  | 11c  NA | Implanted SCS devices can be interrogated to determine subject usage. |
|  | 11d  Table 2 | Changes in analgesic medications are not allowed from time of consent to baseline (for CMM subjects) or SCS device activation (for 10 kHz SCS subjects) so as to not confound the measure of pain scores during this time. Otherwise, all standard of care medical treatments are permitted. |
| Outcomes | 12  P 8, Tables 6 & 7 | The primary endpoint of this study is a composite of safety and effectiveness at 3 months, specifically the percentage of subjects who respond to treatment without a clinically meaningful neurological deficit compared with baseline. A responder is defined as a subject with ≥ 50% reduction in lower limb pain from baseline. For each subject and all analyses, the right and left lower limb VAS scores collected during a single visit will be averaged together to generate a lower limb pain score.  Secondary endpoints:   1. Difference between the treatment groups in proportion of subjects with a lower limb pain VAS score ≤ 3.0 cm at 3 months. 2. Difference between the treatment groups in crossover rates. 3. Difference between the treatment groups in responder rates at 6 months. 4. Difference between the treatment groups in the proportion of remitters (remission is defined as having a lower limb pain VAS score of ≤ 3.0 cm for at least 6 months) at 6 months. 5. Difference between the treatment groups in the proportion of subjects with improvement from baseline in neurological assessment (motor, sensory or reflex) at 3 months. 6. Difference between the treatment groups in the proportion of subjects with overall improvement from baseline in neurological assessment (motor, sensory, reflex) at 6 months. 7. Difference between the treatment groups in changes in health-related quality of life as assessed by the EuroQol Five Dimensions questionnaire (EQ-5D-5L) at 6 months. 8. Difference between the treatment groups in the average percentage change from baseline in HbA_1c_ levels at 6 months.   Tertiary endpoints:   1. Difference between the treatment groups in the average percentage change from baseline in lower limb pain VAS scores at 3 and 6 months. Within group evaluations will be done at 12 and 24 months. 2. Difference between the treatment groups in proportion of subjects with ≥ 30% improvement in lower limb pain VAS at 3 and 6 months. Within group evaluations will be done at 12 and 24 months. 3. Within group evaluation of proportion of remitters at 12 and 24 months. |
|  |  | 1. Within group evaluation of responder rates at 12 and 24 months. 2. Within group evaluation of proportion of subjects with improvement from baseline in neurological assessment (motor, sensory or reflex) at 12 and 24 months. 3. Difference between the treatment groups in Numbers Needed to Treat (NNT) based on responder rates at 3 and 6 months. Within group evaluations will be done at 12 and 24 months. |
|  |  | 1. Difference between the treatment groups in average percentage change from baseline in opioid dosage at 3 and 6 months. Within group evaluations will be done at 12 and 24 months. 2. Difference between the treatment groups in average percentage change from baseline in PDN-specific analgesic dosages at 3 and 6 months. Within group evaluations will be done at 12 and 24 months. 3. Difference between the treatment groups in average percentage change from baseline in HbA_1c_ levels at 3 months. Within group evaluation will be done at 12 and 24 months. 4. Difference between the treatment groups in average percentage change from baseline in diabetic control medication dosages at 3 and 6 months. Within group evaluations will be done at 12 and 24 months. 5. Difference between the treatment groups in average percentage change from baseline in BMI at 3 and 6 months. Within group evaluations will be done at 12 and 24 months. 6. Difference between the treatment groups in the average percentage change from baseline on distance covered during the 6MWT at 3 months. Within group evaluations will be done at 12 and 24 months. 7. Difference between the treatment groups in the change over time in size of lower limb wounds at 3 and 6 months. Within group evaluations will be done at 12 and 24 months. 8. Difference between the treatment groups at 3 and 6 months in health economic outcomes, including: 1) healthcare utilization [i.e. medications, office visits, ER visits, hospital admissions, medical tests, etc.]; 2) employment status; and 3) health-related quality of life as assessed by the EuroQol Five Dimensions questionnaire (EQ-5D-5L) and the Diabetes Quality of Life measure (DQOL). Within group evaluations will be done at 12 and 24 months. |
| Participant timeline | 13  Table 4 | See figure next page |

| **Assessment** | **Enrollment** | | | **Trial & Permanent Phase**  **(SCS only)** | | | | **Follow-up Phase** | | | | | | |
| --- | --- | --- | --- | --- | --- | --- | --- | --- | --- | --- | --- | --- | --- | --- |
| Visit | Consent | Entry Criteria | Baseline Assessment | Trial Implant | End of Trial  (EoT) | Permanent Implant | Device Activation (DA) | 1 Month Visit | 3 Month Visit | 6 Month  Visit | 9 Month  Visit | 12 Month  Visit | 18 Month  Visit | 24 Month  Visit |
| Informed Consent | X |  |  |  |  |  |  |  |  |  |  |  |  |  |
| Medication Usage |  | X | X | X | X | X | X | X | X | X | X | X | X | X |
| Healthcare Utilization |  |  | X |  |  |  |  | X | X | X | X | X | X | X |
| Pain Assessment (VAS) |  | X | X |  | X |  |  | X | X | X | X | X | X | X |
| Weight |  | X | X |  |  |  |  |  | X | X |  | X |  | X |
| Neuropathic Pain Assessment (DN4) |  |  | X |  | X |  |  | X | X | X |  | X |  | X |
| Modified Neuropathy Symptom Score (NSS) |  |  | X |  | X |  |  | X | X | X |  | X |  | X |
| Brief Pain Inventory (BPI-DPN) |  |  | X |  |  |  |  | X | X | X | X | X | X | X |
| Pain Experience (SF-MPQ-2) |  |  | X |  |  |  |  |  | X | X |  | X |  | X |
| Diabetes Quality of Life (DQOL) |  |  | X |  |  |  |  |  | X | X |  | X |  | X |
| Quality of Life Assessment (EQ-5D-5L) |  |  | X |  |  |  |  |  | X | X |  | X |  | X |
| Pain and Sleep Assessment (PSQ-3) |  |  | X |  |  |  |  | X | X | X | X | X | X | X |
| Pt Global Impression of Change (PGIC) |  |  |  |  |  |  |  |  | X | X |  | X |  | X |
| Clin Global Impression of Change (CGIC) |  |  |  |  |  |  |  |  | X | X |  | X |  | X |
| Assessment of Functioning (GAF) |  |  | X |  |  |  |  | X | X | X | X | X | X | X |
| 6-Minute Walk Test (6MWT) |  |  | X |  |  |  |  |  | X |  |  | X |  | X |
| Hemoglobin A1c |  | X |  |  |  |  |  |  | X | X |  | X | X | X |
| Subject Satisfaction |  |  |  |  |  |  |  |  | X | X |  | X |  | X |
| Neurological Assessment |  |  | X |  | X |  |  |  | X | X |  | X |  | X |
| Work Status & Disability |  |  | X |  |  |  |  |  |  | X |  | X |  | X |
| Adverse Event Monitoring |  | X | X | X | X | X | X | X | X | X | X | X | X | X |
| Wound Assessment |  |  | X |  |  |  | X | X | X | X | X | X | X | X |

| Sample size | 14  P 7 | Up to 432 subjects will be screened at multiple clinical sites in the United States. Assuming a 50% screen failure rate, an estimated total of 216 subjects will be randomized, resulting in approximately 108 subjects assigned to each treatment group. The subjects will continue with their respective treatments through the 3 month primary endpoint with an expected 10% attrition rate, resulting in approximately 97 subjects in each group at the primary endpoint. This is the sample size required based on the following assumptions: a 60% responder rate for the 10 kHz SCS group (80% trial success rate and 75% responders at 3 months among permanent implant subjects), a 36% responder rate for the CMM only group, 90% power, and two-sided type I error of 0.05. |
| --- | --- | --- |
| Recruitment | 15  P 6 | Investigators will recruit subjects from their current patient population as well as meet with referring physicians to inform them of the study. Sponsor will provide IRB-approved brochures and posters as well as support social media advertising as needed. |
| **Methods: Assignment of interventions (for controlled trials)** | | |
| Allocation: |  |  |
| Sequence generation | 16a  P 6-7 | The randomization for each site will be performed by a block randomization method developed by an independent statistician. The randomization will be stratified by average baseline pain VAS score and the baseline hemoglobin A1c (HbA1c) level. Thus, there will be 4 strata per site. |
| Allocation concealment mechanism | 16b  P 7 | Concealed allocation achieved via computer assignment of treatment arm with Investigational site staff and study sponsor personnel unaware of block size and randomization list. |
| Implementation | 16c  P 7 | An independent statistician will generate the allocation sequence, Investigational sites will enrol participants, and a computer database will assign interventions. |
| Blinding (masking) | 17a  P 7 | Blinding subjects or investigator teams to the treatment assignment is not feasible due to the nature of the treatments, specifically an implanted medical device compared with CMM. |
|  | 17b | NA |
| **Methods: Data collection, management, and analysis** | | |
| Data collection methods | 18a  P 8 | Data will be collected using eCRFs via an Electronic Data Capture (EDC) system (M-Core, Medrio Inc.). Data will be entered directly into eCRFs in the EDC system at the sites. The clinical site will record data on outcome variables as well as adverse events should they occur. Subject confidentiality will be maintained and each subject will be identified by his or her subject number. Subject names will not be published. |
|  | 18b  NA | Investigational site staff will be trained on importance of subject retention throughout follow-up. |
| Data management | 19  P 8 | Data will be collected using eCRFs via an Electronic Data Capture (EDC) system (M-Core, Medrio Inc.). Data will be entered directly into eCRFs in the EDC system at the sites. The clinical site will record data on outcome variables as well as adverse events should they occur. Subject confidentiality will be maintained and each subject will be identified by his or her subject number. Subject names will not be published. Source documents will be maintained by the Investigator and made available to the Sponsor for the purpose of monitoring the study. Investigators will be required to keep study records for a period of two (2) years or as defined by the local law and regulations where the study is conducted. Passwords will be issued to appropriate personnel to insure confidentiality and protection of data. All original patient files must be stored for the longest possible time permitted by the regulations at the hospital, research institute, or medical practice or at a secure off-site facility. If archiving can no longer be maintained by the site, the investigator will notify the sponsor. All data and documents shall be made available on request of the relevant authorities in case of an audit and/or inspection. The sponsor will archive and retain all essential clinical investigation documents from prior, during and (as specified) after the clinical investigation as per requirements. |
| Statistical methods | 20a  P 8-9 | General considerations:   1. Descriptive statistics will be used to summarize all subject Baseline and outcome data collected during the study. Continuous variables will be summarized using means, standard deviations, medians, and ranges. Categorical variables will be summarized in frequency distributions. 2. Statistical analyses will be performed by validated software (e.g., SAS, IBM/SPSS, or Cytel Software) 3. Statistical tests appropriate to the endpoint being examined will be used and identified. Parametric tests (e.g., Student’s t-tests) will be utilized, if the distributional properties of the data are suitable. If parametric tests are not indicated, the associated non-parametric tests (e.g., Mann-Whitney tests, Fisher’s Exact Tests) will be used. 4. A two-sided p-value of 0.05 or less for the primary endpoint will be considered evidence of statistical significance. Reported p-values for all other tests will be considered nominal and unadjusted for multiple testing, without conclusions regarding statistical significance levels. 5. Copies of databases used to prepare clinical report summaries will be archived to enable any statistical analyses performed to be replicated. |
|  |  | 1. A full data listing will be prepared, including an electronic version in a standard computer-accessible format (e.g., SAS) at the completion of the study. Listings of data represented on the case report forms (eCRF) will be provided for all key baseline, demographic and outcome variables to facilitate further investigation of tabulated values and to allow for clinical review of safety variables.   The primary analysis population for this endpoint is the ITT population. A secondary analysis will be performed in the PP population. The responder rates will be compared between groups with a Fisher’s Exact Test. Secondary analyses will be conducted as described in the Statistical Analysis Plan. |
|  | 20b  NA | The following subgroup results for the primary study endpoint will be examined:   - Results at the 3-month primary assessment in the subgroup randomized to CMM + HF10 but who failed the trial phase and were followed for 6 months under CMM therapy - Follow-up results in those subjects who crossed over to the other treatment group after the initial 6-month visit   Additional exploratory analyses may be performed to examine the consistency of results in selected subgroups (e.g., based on gender, study site, age, pain duration, pain severity, glycemic control, etc.). These analyses may also take the form of multivariable analyses, where the contributions from membership in multiple subgroups to a study endpoint are simultaneously estimated. |
|  | 20c  P 9 | The following analysis populations are defined for the study:  Intention-to-Treat (ITT): All subjects randomized into the CMM and CMM+HF10 study groups. This is considered the Safety Population for purposes of reporting on any reported adverse events.  Per Protocol (PP): All randomized subjects who are either randomized into the CMM group or are randomized to the CMM+HF10 group and receive a SENZA System implant, and who complete the 3-month primary assessment. |
| **Methods: Monitoring** | | |
| Data monitoring | 21a  P 9-10 | A Clinical Events Committee (CEC) will serve in an advisory role to review safety data at interim points during the study, including the review of adverse events (AEs) and adjudication of the relatedness and seriousness of serious adverse events (SAEs). If needed, the CEC will also review unanticipated serious adverse device events on an urgent basis. The CEC is responsible for oversight of study safety considerations and will make recommendations to sponsor based on their reviews. |
|  | 21b  P 7 | A single interim analysis will be performed to reassess sample size assumptions for evaluation of the primary study endpoint when 25% of the subjects have completed the 3-month primary assessment. This interim analysis will be performed by an independent third party, and knowledge of specific study results will be kept blinded from the sponsor and other study participants. The interim analysis will evaluate the conditional power to detect a significant difference at the completion of the study between treatment groups, given the available 3-month data. A recommendation regarding sample size will be made to the sponsor based on the estimated conditional power:  Conditional power ≤ 20%: very unfavorable, stop the study early for futility  Conditional power > 20% and ≤ 40%: unfavorable, but no increase in sample size  Conditional power > 40% and ≤ 80%: promising, increase the sample size by an estimated amount to restore the original design power level of 90%  Conditional power > 80%: favorable, but no increase in sample size  The above conditional power intervals result in three possible recommendations that may be made to the sponsor: stopping early for futility, no change in sample size, and increasing the sample size. The sponsor reserves the right to accept or reject any of these recommendations.  A recommendation for no change in sample size associated with “unfavorable” or “favorable” interim results helps ensure the blinding of the actual results. A recommendation to increase the sample size signifies “promising” results, but the results may be in a relatively wide conditional power interval (40% - 80%), and are, similarly, not revealed.  Since there is no provision for stopping the study early on the basis of positive interim findings, there is no impact on the final alpha level (two-sided, alpha = 0.05) for the evaluation of the primary endpoint. |
| Harms | 22  P 9-10 | Subjects will be assessed for adverse events starting at enrollment and continuing through study completion. If an adverse event occurs, an adverse event eCRF will be completed. The event will be followed until resolution or determination that the subject’s condition is stable.  The Investigators shall categorize all adverse events for seriousness, severity, and relationship. All determinations of severity, device relation, and resolution are made by the Investigator and not by the Sponsor.  For purposes of consistent adverse event reporting and analysis, adverse events will be categorized using the Medical Dictionary for Regulatory Activities (MedDRA®) terminology, consistent with the *MedDRA® Term Selection: Points to Consider* document. Events are grouped by System Organ Classification (SOC) and Preferred Term (PT), according to diagnosis and/or event description as provided by the Investigator on the eCRF.  All AEs, SAEs, and UADEs (see list in section C.1) occurring during the study will be collected. The site will document onset, severity, treatment/intervention provided, relationship to the treatment/procedure, and resolution and record the data on the Adverse Event eCRF. Any UADEs and/or deaths occurring during the study procedures will also be evaluated to determine whether the SCS system or medication might have caused or contributed to the event.  All SAEs/UADEs will be documented and reported to Nevro as soon as possible, but no later than 48 hours after becoming aware of the SAE.  The Investigator or site staff may report an event to the sponsor by email, telephone or fax initially, but must follow-up by completing an Adverse Event eCRF. The eCRF should be accompanied by copies of source documentation regarding the event (e.g., physician/nurse notes or summaries/hospitalizations records; including hospital admission reports, progress notes and hospital discharge notes). The Investigator must also report the SAE to the IRB according to their local regulations. In the event of a subject death, all available information (e.g. autopsy or other post-mortem findings) should be provided with or on the eCRF.  An Investigator shall submit a report to the sponsor of any UADE occurring during the study as soon as possible, but in no event later than 10 working days after the Investigator first learns of the effect. Nevro will conduct an evaluation of the event to determine whether the event is an anticipated event based on labeling and risk analysis. The Investigator must also report the event to the IRB if it is determined to be unanticipated. |
|  |  | All AEs will be followed until the event is resolved (with or without sequelae). If a device-related event is ongoing at the time of study completion or termination, the subject will be followed until resolution or the Investigator determines that the subject’s condition is stable.  In addition to the above reporting and in accordance with Medical Device Reporting requirements of 21 CFR 803, when using commercial medical devices, device related SAEs and UADEs must be reported to Nevro’s Product Support Department at the above email, phone number, or fax number, as specified in the manufacturer’s labeling/manuals. If the “study related” box is checked on the AE eCRF, this reporting applies.  For all treatments administered as CMM, the Investigator shall report suspected medication-related or treatment-related AEs to the FDA or manufacturer according the site’s usual practice and/or the manufacturer’s label. |
| Auditing | 23  NA | Sponsor will regularly visit Investigational sites to monitor trial conduct. For this study, Site Initiation Visit, Interim Monitoring Visits and Close-Out Visit will be performed in accordance to the applicable Nevro SOPs, the protocol and Monitoring Plan. At a minimum, the site will be monitored within four weeks after the first subject has completed the baseline visit. Once enrolling, the site must be monitored at least every 6 months. If no enrollment; the site will not be routinely monitored and a study close out visit could be scheduled within 6 months of Study Initiation. |
| Ethics and dissemination | | |
| Research ethics approval | 24  P 6 | Enrollment of subjects will occur at the clinical sites only after IRB approval of the protocol, consent form, and any subject-facing materials has been obtained. |
| Protocol amendments | 25  NA | Changes to the protocol will be communicated to Investigational sites via notes to file with IRB notification when required. |
| Consent or assent | 26a  NA | Investigational site staff will perform and document the informed consent process. |
|  | 26b | NA |
| Confidentiality | 27  P 8 | Subject identity will remain as confidential as possible under federal, state and local law. The information from study records will be made anonymous (subject name will be replaced by a study identification number) when entered onto separate forms that are sent to the study sponsor (Nevro Corp.) and when the study data is processed by computer. Additionally, subject name will not be used in any publication or presentation. |
|  |  | The original records and the study data forms will be stored in the study doctor’s office. Research staff at the study doctor’s office will enter data into a secure database and identified only by the study subject identification number. The study doctors and the sponsor’s research staff also have the right to consult the research files within the context of the study.  For auditing purposes, competent public health authorities (including local and state enforcement agencies, the Food and Drug Administration (FDA), and other federal authorities may have access to study records. Additionally, representatives of the Institutional Review Board (IRB) may also review study records |
| Declaration of interests | 28  P 11 | Investigators will disclose any conflicts of interest when presenting conference data and submitting for publication. |
| Access to data | 29  NA | Sponsor will have access to the complete data set with right to publish overall trial results prior to any individual site publication. |
| Ancillary and post-trial care | 30  NA | Subjects with an implanted device will continue to be supported by Nevro after trial completion, similar to patients with commercial implants. There are no plans to compensate subjects for study-related injury. |
| Dissemination policy | 31a  NA | Study data will be communicated at medical conferences, in peer-reviewed journal publications, and listed on clinicaltrials.gov. The sponsor has priority to publish the main findings prior to individual site publications. |
|  | 31b  NA | Authorship eligibility will follow the International Committee of Medical Journal Editors (ICMJE) recommendations. |
|  | 31c | NA |
| Appendices |  |  |
| Informed consent materials | 32 | Template consent form follows on the next page. |
| Biological specimens | 33 | Blood samples will be collected to measure hemoglobin A1c according to standard laboratory practices. There is no plan for storage or further analysis of samples. |

*It is strongly recommended that this checklist be read in conjunction with the SPIRIT 2013 Explanation & Elaboration for important clarification on the items. Amendments to the protocol should be tracked and dated. The SPIRIT checklist is copyrighted by the SPIRIT Group under the Creative Commons “[Attribution-NonCommercial-NoDerivs 3.0 Unported](http://www.creativecommons.org/licenses/by-nc-nd/3.0/)” license.

*Nevro Corp.*

*CA2016-5 US*

| **Research subject information and Consent form** | |
| --- | --- |
| **TITLE:** | A Post-Market, Multicenter, Prospective, Randomized Clinical Trial Comparing 10 kHz Spinal Cord Stimulation (HF10™ Therapy) Combined with Conventional Medical Management to Conventional Medical Management Alone in the Treatment of Chronic, Intractable, Neuropathic Limb Pain |

**This consent form contains important information to help you decide whether to participate in a research study.**

The study staff will explain this study to you. Ask questions about anything that is not clear at any time. You may take home an unsigned copy of this consent form to think about and discuss with family or friends.

- **Being in a study is voluntary – your choice.**
- **If you join this study, you can still stop at any time.**
- **No one can promise that a study will help you.**
- **Do not join this study unless all of your questions are answered.**

**After reading and discussing the information in this consent form you should know:**

- Why this research study is being done;
- What will happen during the study;
- Any possible benefits to you;
- The possible risks to you;
- Other options you could choose instead of being in this study;
- How your personal health information will be treated during the study and after the study is over;
- Whether being in this study could involve any cost to you; and
- What to do if you have problems or questions about this study.

**Please read this consent form carefully.**

**Research subject information and Consent form**

**Title:** A Post-Market, Multicenter, Prospective, Randomized Clinical Trial Comparing 10 kHz Spinal Cord Stimulation (HF10™ Therapy) Combined with Conventional Medical Management to Conventional Medical Management Alone in the Treatment of Chronic, Intractable, Neuropathic Limb Pain

**Protocol No.:** CA2016-5 US

WIRB^®^ Protocol #20171535

**Sponsor:** Nevro Corp.

**Investigator:** Name

Address

City, State Zip

Country

**STUDY-RELATED**

**PHONE NUMBER(S):** Name

Phone number (24-hour number required)

**INTRODUCTION**

You are being invited to take part in this research study to evaluate the safety and effectiveness of an electrical nerve stimulation device called a spinal cord stimulator (SCS) because you have pain in the lower limbs.

SCS involves the surgical placement of two leads (which look like very thin wires) into a small area near your spinal cord. Electrical stimulation is delivered through these wires, in an attempt to provide pain relief.

The SCS system used in this study is the Senza System, made by Nevro Corp. The device is a small, battery-operated, rechargeable SCS with an attached electrode. The Senza System has been approved by the Food and Drug Administration (FDA).

The purpose of this study is to compare electrical stimulation of the spinal cord using the Senza system combined with standard medical treatments to standard medical treatments alone for chronic (long-term) pain of the lower limbs due to painful diabetic neuropathy.

**ABOUT THIS STUDY/NUMBER OF PEOPLE WHO WILL PARTICIPATE**

Your participation in this study is completely voluntary. Up to 432 subjects at multiple centers in the United States will take part in this study.

If you are part of the study, your participation is likely to be about 26-32 months and requires about 14 clinic/doctor’s office visits, including 0-2 surgical visits in a clinic or hospital. You will be randomly assigned to one of two treatment groups. Similar to flipping a coin, instead of “heads” or “tails,” you will be assigned to either: 1) continue with your current treatments with optimization at the discretion of your physician, or 2) continue with your current treatments with the addition of SCS. After 6 months in the study, if you qualify, you may choose to cross over to the alternative treatment arm.

There may be reasons why you cannot participate in this study. Your study doctor will discuss these with you.

If you decide to participate and sign this consent form, you will undergo the tests and procedures described below.

**SCHEDULE OF STUDY VISITS**

**Enrollment and Eligibility/Baseline Assessment (2-3 clinic visits)**

Your first visit will last about an hour and a half. Your doctor and/or study staff will explain this study to you and you will be asked to sign this consent form before you may participate in this study. You will have some tests such as a physical examination and a blood test. You may be sent to a lab located near your doctor’s office for this blood test. If you are a woman of child bearing potential, you will be asked to have a urine pregnancy test. A psychological evaluation, magnetic resonance imaging (MRI) and other tests may also be scheduled. The study doctor and research staff will review your medical and surgical history and ask about the medications you are taking. You will fill out a questionnaire about your pain levels. You will be asked to attend any scheduled tests prior to your next clinic visit.

At the next visit, your study doctor will go over your initial test results and tell you if you qualify for the next step of the study called the Baseline Assessment. If you are not eligible, you will be discontinued from the study.

The Baseline Assessment will take about an hour and a half. During this visit, your doctor will perform a neurological test. You will be asked to complete several questionnaires about your pain levels, what your pain feels like, how you manage daily life, the functioning of your lower limbs, and your sleep patterns. You will be assessed for how far you can walk in 6 minutes. Research staff will review your current medications, ask about your work status, ask about recent visits to other doctors, hospitals, and medical tests you have completed. If you have any lower limb wounds, photographic images will be taken at this visit and at all follow-up visits during this study to document wound healing. Following this visit, research staff will make an appointment for your trial surgery (if assigned to the SCS treatment group) or your next study visit (if assigned to the non-SCS treatment group).

**Trial Surgery and Assessment (1 clinic/hospital visit and 1 clinic visit)**

This section only applies if you are assigned to the SCS treatment group or if you choose to switch to the SCS treatment group after 6 months in the study.

As is normal in SCS therapy, you will first undergo trial stimulation to see if this type of therapy can be helpful for your type of pain. Prior to the procedure, research staff will review your current medications with you and ask you to indicate where your pain is on a diagram. You may also be asked to indicate where additional symptoms, such as numbness, cold, tingling, or burning, are on a diagram. During surgery at a clinic or hospital, your study doctor will place two leads, which look like very thin wires with electrodes on them, near your spinal cord and will then connect them to a temporary trial stimulator that is located outside your body and worn on a belt near your stomach.

The surgery may be performed with a combination of pain medication, sedation, and local and/or general anesthesia.

Fluoroscopy and/or X-ray imaging will be performed to make sure the leads are in the correct place. Photographic images of the surgical site(s) may be taken at this visit or at any time during this study to document surgical procedures and techniques, incision location, lead placement and wound healing, or for other reasons, at the direction of your doctor. The stimulator will be programmed with help from a representative of Nevro Corp., to deliver electrical stimulation through the leads. The stimulation settings will be based on where you have pain and how much pain you have.

Your study doctor will determine when you may leave the hospital, but you will likely leave the same day. Before you leave, you will be given instructions on how to care for your surgical wounds and the trial stimulator. You will also be shown how to work the trial stimulator and the remote that controls the stimulator.

Within 14 days after the surgery, you will return to the clinic for a neurological test and you will be asked to fill out a questionnaire about your pain levels. You will be asked about the medications you are taking and questions about what your pain feels like. You may be asked to record the distribution of pain and additional symptoms, such as numbness, cold, tingling, or burning, on a diagram. Your doctor will take an x-ray to check the position of the leads. If you have enough pain relief as determined by your study doctor, the leads will be removed and you can choose to receive a permanent SCS system. This permanent SCS system will replace the temporary, trial stimulator that was outside your body, with a stimulator that will be implanted in you. If you do not have enough pain relief as determined by your study doctor, the leads will be removed and you will continue with conservative treatments. If this occurs, your study doctor will discuss appropriate treatments with you and perform the assessments listed below for the 1, 3, and 6 month visits before your participation in the study ends. If you were initially assigned to the non-SCS treatment group but crossed over after your 6 month visit and did not have enough pain relief as determined by your study doctor, clinic staff will follow-up with you via telephone about two weeks after your leads are removed to ensure you are doing well. Your participation in the study will end at this point.

**Permanent System Surgery and Device Activation (1 clinic/hospital visit and 1 clinic visit)**:

This section only applies if you are assigned to the SCS treatment group or if you choose to switch to the SCS treatment group after 6 months in the study.

The permanent system surgery will occur within 60 days from the end of the trial stimulation period. Prior to the procedure, research staff will review your current medications with you and ask you to indicate where your pain and additional symptoms, such as numbness, cold, tingling, or burning, are on a diagram. During surgery at a hospital or clinic, your study doctor will either continue to use the two leads that were placed during the trial stimulation (if they were not removed) or place two new leads and connect them to a stimulator that will be implanted in your body, usually in the lower back area, above the buttocks or in the abdomen. The stimulator is small - about 2.7 inches by 1.9 inches by 0.5 inches.

The surgery may be performed with a combination of pain medication, sedation, and local and/or general anesthesia.

Fluoroscopy and/or X-ray imaging will be performed during the surgery to make sure the leads are in the correct place. Photographic images of the surgical site(s) may be taken at this visit or at any time during this study to document surgical procedures and techniques, incision location, lead placement and wound healing, or for other reasons, at the direction of your doctor. At the end of this procedure, no part of your device will be outside of your skin; it will be completely implanted.

Your study doctor may prescribe pre-surgery antibiotics to help prevent infection and additional medications to help with after-surgery pain. Your study doctor will determine when you may leave the hospital, but you will likely leave the same day. Before you leave, you will also be told how to care for your surgery wounds.

The Device Activation visit will last about an hour, and your permanent stimulator will be turned on. It is possible that you may be able to complete the Device Activation on the same day as your surgery. If not, you will need to return a few days after your surgery. The stimulator will be programmed with help from a representative of Nevro Corp. The settings will be based on where you have pain and how much pain you have. You will also be asked some questions about how the stimulation feels.

You will be shown how to use the stimulator's charger and the remote that controls the stimulator. You will also be shown how to change programs and turn the stimulation on and off. If you have any lower limb wounds, photographic images will be taken to document wound healing.

**1 Month, 9 Month, and 18 Month Visits (3 clinic visits)**

You will return to the clinic for scheduled visits at 1 month, 9 months, and 18 months. These visits will be about 1 hour long.

During these visits, you will be asked to fill out questionnaires about your pain levels, how you manage your daily life, the functioning of your lower limbs, and your sleep patterns. You will be asked about the medications you are taking, recent medical appointments and tests, and questions about what your pain feels like. If you have any lower limb wounds, photographic images will be taken to document wound healing. You will be asked about any side-effects you may have experienced. Inform your study doctor of any changes to your medications or overall health. At the 18 month visit, your blood will be drawn for a lab test. You may be sent to a lab located near your study doctor’s office for this blood test.

If you have an implanted stimulator, you will also be asked some questions about how the stimulation feels and if needed, stimulator programming changes may be made with help from a representative of Nevro Corp. Your doctor may take an x-ray to check the position of the leads.

**3 Month, 6 Month, 12 Month, and 24 Month Visits (4 clinic visits)**

At 3 months, 6 months, 12 months, and 24 months into the study you will return to the clinic for scheduled visits that will take about an hour and a half.

During these visits, a neurological test will be done. You will be asked to fill out questionnaires about your pain levels, how you manage daily life, the functioning of your lower limbs, your sleep patterns, your opinion on any change in your overall quality of life and your level of satisfaction with your therapy. Your blood will be drawn for lab tests. You may be sent to a lab located near your study doctor’s office for this blood test. You will be asked about the medications you are taking, recent medical appointments and tests, and questions about what your pain feels like. If you have any lower limb wounds, photographic images will be taken to document wound healing. You will be asked about any side-effects you may have experienced. Inform your study doctor of any changes to the medications you are taking. At 3, 12, and 24 months you will be assessed for how far you can walk in 6 minutes.

If you have an implanted stimulator, you will also be asked some questions about where you have pain and other symptoms, such as numbness, cold, tingling, or burning, and about how the stimulation feels. If needed, stimulator programming changes may be made with help from a representative of Nevro Corp. Your doctor may take an x-ray to check the position of the leads.

For all study patients, with or without a stimulator: After the 24 month visit, your participation in the study will be complete but you will still see your study doctor for any concerns or issues that you might have.

At the end of the study, if you have an implanted stimulator, your stimulator will remain implanted, unless you choose to have it removed, your condition has not improved, or your study doctor decides you should not keep it.

If it is decided that you will not keep the implanted device, your doctor will surgically remove the leads and stimulator at a clinic or hospital. There is a chance that leads may not be removed if your study doctor believes that lead removal may cause tissue injury. Your study doctor will determine when you may leave the hospital but you will likely leave the same day. Before you leave, you will also be told how to care for your surgery wounds. Your doctor or clinic staff will call you 7 to 10 days after the surgery to see if you are having any problems relating to the device removal. If you are not having any problems, your participation in the study is complete. If you are having problems, you will be followed until you have recovered or the study doctor has determined that your condition is stable.

If it is decided that you will keep the implanted device, you will receive additional instructions on how to care for your device and information on any follow-up medical care that will done by your study doctor until the stimulator is removed or you no longer wish to receive therapy.

For safety reasons, when you are participating in a research study, it is important to attend all scheduled visits with your doctor. If you feel you cannot attend all of the study visits listed above, you should not participate in this study.

**Other Tests, Visits and Communications**

At any time during the study, if you experience pain, are not feeling well or are having any problems, please contact your study doctor or clinic staff. Discuss any changes in your health or medications with your study doctor.

If you are not having adequate pain relief at any time during the study you may be asked to return to the doctor’s office/clinic for an extra study visit(s) of about an hour to change the settings of your stimulator, if applicable, or adjust other treatments, to give you better pain relief. If needed, additional testing may also be done. An x-ray may be taken at any time during the study to see the position of the leads if you have an implanted stimulator. If your doctor feels that your leads are not in the correct place to give you good pain relief, they may be moved to a different location on your spine.

Throughout the study, you will be contacted by study staff to see how you are doing and remind you of upcoming study visits.

If you have an implanted stimulator, you or your personal representative will also be contacted by Nevro Corp. employees and/or Nevro Corp’s third party contractors (“Nevro Corp. personnel”) throughout the study to see how you are doing, facilitate adjustments to your stimulation, check if you are having problems with the equipment and to answer questions related to the use of the device. Please check the boxes below to indicate how you would like Nevro Corp. personnel to contact you throughout the study and provide the required information on the line after the checkbox. You may check as many boxes as you would like. Standard message and data rates will apply.

□ Call – Home Phone ( ) -

□ Call – Cell Phone ( ) -

□ Text Message ( ) -

□ E-mail @

All medically related matters should be discussed with your study doctor or research staff. If you have any questions about your pain relief, please contact your study doctor or research staff.

**Medication Usage:**

- For patients assigned to receive SCS treatment: You will be required to continue to take the types and doses of pain medications you were taking at the Baseline visit until the Device Activation Visit. This means that if you pass the Trial Phase and receive a permanent stimulator, you will continue on the same medications until the permanent stimulator is turned on.
- For patients assigned to receive SCS treatment: At the time of the trial and permanent implant surgeries, your study doctor may prescribe pre-surgery antibiotics to help prevent infection and additional medications to help with after-surgery pain. These medications are separate from the medications you are already taking. Please follow the directions of the doctor when taking these medications.
- If you are in more pain than you can tolerate at any time during the study, please contact your study doctor who may recommend over-the-counter medicines for immediate pain relief. The study doctor may also choose to prescribe other pain medications.
- You will be required to use the same types and doses of pain medications for two weeks before any scheduled follow-up visit. This is so that your study doctor can determine whether the therapy is working for you.
- You should not increase any pain medication doses or begin taking new pain medications without first consulting with your study doctor.

**POTENTIAL BENEFITS OF YOUR PARTICIPATION**

It is possible that you will not benefit at all from participating in this study. Although the treatments used in this study have been shown to reduce pain in some people with lower limb pain, there is no guarantee of pain relief. Every effort will be made to find appropriate treatments that will provide you with pain relief, but Nevro Corp. makes no guarantee that you will receive pain relief. If you experience pain relief, it is not known how long the benefit might last.

**POTENTIAL RISKS OF YOUR PARTICIPATION**

If you are treated by any doctor or healthcare provider not associated with this study, immediately inform them that you are a trial patient. If you fail to do so, it could put you at significant risk.

Your study doctor will follow the standard of care to administer conventional medical treatments for your condition. Treatments may include, but are not limited to, medications, physical therapy, cognitive therapy, chiropractic care, nerve blocks, and other non-invasive or minimally invasive therapies. The risks associated with these treatments are typical of routine medical care.

The risks of drawing blood from a vein include discomfort at the site of puncture; possible bruising and swelling around the puncture site; rarely an infection; and, uncommonly, faintness from the procedure. Throughout the study a total of approximately 3 tablespoons of blood will be taken.

If you are assigned to receive SCS treatment as part of this study: There are a number of known risks associated with SCS procedures, devices and therapy which are listed below. Additionally, you might experience side effects or complications that are not listed below. Some side effects of trial participation are not yet known and not every risk or side effect can be predicted. Tell your study doctor right away if you have any problems.

Some of the known risks of SCS treatment are:

| **Type of Risk** | **Level of Risk** | **Description of Risk** |
| --- | --- | --- |
| Implant Procedure | Possible | Surgical complications, infection, cellulitis (bacterial infection of the skin), abscess (pus that causes swelling), fever, sepsis (blood infection) |
|  |  | Poor lead placement or migration requiring removal or repositioning |
|  |  | Bleeding |
|  |  | Cerebrospinal fluid leak (leaking of fluid that surrounds the brain and spinal cord) |
|  |  | Epidural hemorrhage (collection of blood just outside the brain or spinal cord) |
|  |  | Temporary pain or persistent tenderness/pain at implant site |
|  |  | Inadequate wound healing |
|  |  | Hematoma (collection of blood outside blood vessels), seroma (pocket of clear fluid that happens after surgery) or thrombosis (formation of a blood clot inside a blood vessel) |
|  |  | Risks associated with anesthesia |
|  | Unlikely | Nerve/nerve root/spinal cord injury |
|  | Very Unlikely | Death  Paralysis |
| Stimulation | Possible | Increased pain (other than at implant site or areas being treated) |
|  |  | Increased pain (higher than before device implant) in the areas being treated |
|  |  | Loss of pain relief, unpleasant paresthesia |
|  |  | Undesirable sensation |
|  |  | Undesirable/unwanted stimulation due to cellular changes around electrodes, changes in electrode position, loose electrical connections, or lead failure |
|  |  | Uncomfortable stimulation of tissue around the leads including skin and muscle |
|  |  | Intermittent stimulation |
|  |  | Tingling, prickling or numbness in pain area |
|  | Unlikely | Malfunction |
|  | Very Unlikely | Seizure |
| Implanted Device | Possible | Tissue reaction or allergy to implanted materials |
|  |  | External sources of electromagnetic interference that cause the device to malfunction and could affect stimulation |
|  | Unlikely | Persistent pain at implant site (electrode or IPG) |
|  |  | Failure of device components or the battery including lead breakage or movement (migration), hardware malfunctions, loose connections, electrical shorts or open circuits and lead insulation breaches |
|  |  | Failure or malfunction requiring removal and re-implantation |
|  | Very Unlikely | Skin erosion over lead or IPG site |
|  |  | Pressure sores |
| External Device | Unlikely | Malfunction |
|  |  | Uncomfortable heating effects, discomfort or burn |

**PRECAUTIONS AND WARNINGS FOR SCS TREATMENT**

The safety of spinal cord stimulation during pregnancy has not been established, and it is possible that harmful side effects could occur to both the mother and unborn child. For this reason, if you are pregnant you cannot participate in the study. If you are a woman of child bearing potential, you should use effective forms of contraception (birth control) while participating in this study. If you become pregnant during the study, your participation in this study will end. If you become pregnant during the study, tell your doctor immediately. It is not known if spinal cord stimulation while nursing is safe. If you are nursing talk to your doctor about possible risks.

There may be unforeseen events that occur during this trial that would result in you being removed from the study. You will be informed by your study doctor or clinic staff if your participation in the trial needs to end.

The stimulator may affect the operation of other implanted devices such as a cardiac pacemaker. You should advise your study doctor if you have another active (a device that has an electrical energy source) implant.

Changes in your posture (such as stretching, twisting, or bending) or quick movements may alter the stimulation feeling and may make it unpleasant. If you experience unpleasant sensations from your stimulator, your study doctor will explain to you how to adjust the device settings to avoid these sensations. Within the first weeks after implant surgery, your body is still healing and you should not make forceful or sudden movements, as it may change the position of the implanted leads, making the SCS less effective in relieving your pain. If that occurs, the lead may have to be repositioned through another surgery.

To avoid possibly damaging the stimulator due to increased pressure, you should not scuba dive or use a hyperbaric chamber while the stimulator is in your body.

While you have the leads or stimulator in your body, you should not receive shortwave diathermy (therapy that creates heat deep in the body), microwave diathermy, or therapeutic ultrasound (procedures that use sound waves to treat painful muscles) at any time while the stimulator is implanted. The use of diathermy following implant may cause serious injury or death.

Additionally, medical or hospital procedures such as external defibrillation, lithotripsy (shock waves used to break up kidney or gallstones), high-output ultrasounds, electrocautery, radiation therapy, and ultrasonic scanning could disrupt the function of the stimulator or cause unwanted or unpleasant stimulation (jolts). Before undergoing any such procedures, please inform medical personnel that you have an implanted SCS stimulator and leads.

Exposure to strong magnetic fields or some security screening devices, such as theft detection systems, airport security scanners, security wands, power lines/generators or arc welders could also cause you to experience unpleasant sensations.

Magnetic resonance imaging (MRI) could result in heating of tissue and subsequent tissue damage or serious injury. You should not have magnetic resonance imaging (MRI), with stimulation on or off, unless your study doctor has determined it is safe for you to do so based on the approved labeling of the implanted device. MRI may be considered more sensitive than computed tomography (CT) in detecting abnormalities in the different soft tissues of the body and therefore could be especially useful in imaging the brain, muscles, the heart, and cancers. This might be important to your health, so if you have any concerns your study doctor can discuss any of these procedures with you and answer questions you may have.

Once placed in your body, you should avoid trying to rub or touch the stimulator below your skin. These actions may cause the stimulator to twist or rotate (making it difficult to charge the stimulator) and may also cause your skin above the stimulator to get thinner.

If your stimulation causes a tingling sensation, you should not drive motorized vehicles or work with potentially dangerous machinery or equipment with the stimulation on, as the stimulation may distract you. You will need to turn the stimulation off to do such activities.

If you experience discomfort or excessive redness around the wound areas, you may need to be examined for infection. If you have a severe infection, your device may have to be removed. You should not use the charger if the incision from surgery is not healed, because the charging pad and charger are not sterile.

The charger may become warm during charging. The device should not be charged while you are sleeping. The charger should not be used over areas of the skin that are not sensitive to the feeling of warmth. Only the manufacturer’s charging accessories should be used while charging. Failure to follow these instructions may result in a burn. If you experience any discomfort or pain, stop charging.

This research study involves exposure to radiation through x-rays and fluoroscopy and exposure to radiation can increase your risk of developing cancer. You will have at least three x-ray or fluoroscopy imaging procedures while being on this study, but you may need more as determined by your doctor. These procedures use only small amounts of radiation, but exposure to radiation accumulates over your lifetime and should be kept as low as possible. Radiation exposure is just one factor among many that can increase your risk of developing cancer. Other factors associated with cancer development are advanced age, family history (genetics) and lifestyle factors (smoking, diet, exercise, etc.). Tell your study doctor now if you have been in other research studies where you had ionizing radiation. Also tell your study doctor if you have been exposed to radiation in other ways, like on your job or in radiation therapy.

**ALTERNATIVE TREATMENT**

If you choose not to be in the study, or decide to stop participating during the study, you can talk with your study doctor about other treatments for your pain condition. These include medications, physical therapy, other commercial SCS devices, transcutaneous electrical nerve stimulation (TENS), and surgical procedures. Your study doctor will discuss all your options with you and answer any questions you may have.

**VOLUNTARY PARTICIPATION AND WITHDRAWAL FROM THE STUDY**

Your participation in this study is voluntary. If you choose not to participate, there will be no penalty or loss of benefits which you would otherwise receive. If you agree to participate and then change your mind, you are free to withdraw your consent and stop your participation at any time. If your study doctor discovers changes in your overall health, you will be informed in a timely manner. Your decision to withdraw from the study will not affect your ability to receive medical care for your condition and you will not have any penalty or lose any benefits which you would otherwise receive.

If you choose not to continue in the study after you received SCS treatment with the Senza System, the stimulator and leads will be surgically removed and you will have to return all device parts that you were given (including the remote control, the external stimulator and the charger).

There is a chance that the leads may not be removed if your study doctor believes that lead removal may cause tissue injury.

You may also be withdrawn from the study with or without your consent, and possibly be asked to have the device taken out, for one or more of the following reasons:

- If your study doctor decides that continuing your participation could be harmful to your health.
- If the study is stopped by Nevro Corp. (the study sponsor), the FDA or the Institutional Review Board.
- You do not consent to continue in the study after being told of changes in the research that may affect you.
- Other administrative reasons or unanticipated circumstances.

Your study doctor will be available to answer any questions regarding your participation in the study. If you leave the study before the planned final visit, you may be asked by the study doctor to have some of the end of study procedures done.

**CONFIDENTIALITY**

Your doctor will collect medical information about you (for example, medical histories, insurance and billing information, and results of any tests, examinations or procedures you undergo while in the study). If you consent to be in this study, you will be allowing the investigators to use and disclose your personal health information for purposes of the research, publication, and/or teaching. The following people will have access to your study records:

- Study site personnel
- Authorized representatives of Nevro Corp.
- Representatives of Regulatory Authorities, such as the FDA and the Department of Health and Human Services (DHHS)
- Representatives of the Institutional Review Board (IRB)

Your identity will remain as confidential as possible under federal, state and local law. The information from your study records will be made anonymous (your name will be replaced by a study identification number) when entered onto separate forms that are sent to the study sponsor (Nevro Corp.) and when the study data is processed by computer. Additionally, your name will not be used in any publication or presentation.

The original records and the study data forms will be stored in the study doctor’s office. Research staff at your study doctor’s office will enter your data into a secure database and you will be identified only by your study identification number. The study doctors and the sponsor’s research staff also have the right to consult the research files within the context of the study.

For auditing purposes, competent public health authorities (including local and state enforcement agencies, the Food and Drug Administration (FDA), and other federal authorities may have access to your study records. Additionally, representatives of the Institutional Review Board (IRB) may also review your study records.

If your personal health information is given to those who are not required to comply the Health Insurance Portability and Accountability Act (HIPAA), your Protected Health Information will no longer be protected by this law and could possibly be used or disclosed in ways other than those listed above.

By signing this form, you authorize the study doctor to use and share your information as described above. If you later change your mind about sharing your information, you may revoke (take back) this authorization by writing to the study doctor. This authorization will never expire unless and until you revoke this authorization. If you revoke this authorization, you will not be allowed to continue your participation in this study.

You have the right to look at any of your collected study information and can request to have corrections made except where data must remain confidential to maintain the integrity of the study.

E-mail and Text Message Confidentiality

E-mail and text messaging allows you and Nevro Corp. personnel to contact each other efficiently, but is not a completely secure means of communication. E-mail and text messages can be addressed to the wrong person and can be accessed by others while being sent/received and while being stored on your device. Your consent to receive e-mails and/or text messages is not required in order to receive treatment and your decision will not affect your health care in any way.

**DURATION OF PARTICIPATION**

Your participation in this research study is expected to last approximately 26-32 months.

**COSTS**

Neither you, nor your insurance company will be billed for the Senza device or for any of the procedures that are performed specifically related to the device in this study.

You and/or your insurance company will however be billed for all treatments you undergo other than SCS treatment, including the cost of any procedures, treatments, and medications related to your care that are considered normal and customary practice. For such routine costs, you may be responsible for any co-payments or deductibles required under your insurance.

In addition, you and/or your insurance company will be billed for the replacement, revision or explant of the Senza System should these procedures be required after your participation in the study has ended.

**PAYMENT FOR PARTICIPATION**

You will receive payment for each group of study visits to offset your time and personal expenses. You will not receive any other form of payment.

Below is a summary of the payment for participation:

| **Visits** | **Payment to You** |
| --- | --- |
| Enrollment Eligibility/Baseline Assessment | $100.00 |
| Trial Surgery, Permanent Surgery, Device Activation (SCS treatment group only) | $100.00 |
| 1 Month Visit, 3 Month Visit, 6 Month Visit | $150.00 |
| 9 Month Visit, 12 Month Visit | $100.00 |
| 18 Month Visit | $100.00 |
| 24 Month Visit | $100.00 |
| Total for Completing All Study Visits | $650.00 |

If you do not finish the study, you will only be paid for the last group of completed visits.

Nevro Corp. may patent or sell discoveries that result from this research. Nevro Corp. or your study doctor will not compensate you for the development of any products based on this study. You do not have rights to future inventions.

**TREATMENT AND COMPENSATION FOR ILLNESS/INJURIES**

If you are injured as a result of participating in this study, treatment will be provided or arranged by your study doctor.

There are no plans to provide monetary compensation for the cost of treating study related injuries and no non‑medical compensation by Nevro Corp. or your doctor.

The study doctor, hospitals and institutions where they work will assume no responsibility for any illness or injury to you as a result of your being in this study. However, the institution or hospital may be responsible if any illness or injury is caused by an error (negligence) on the part of the institution, its employees, or agents. You still have the right to bring a lawsuit if you think you were harmed and deserve compensation.

**ADDITIONAL INFORMATION**

You will be notified of any new information that is learned during the course of this study that may affect your health, safety or willingness to participate.

A description of this clinical trial will be available on [http://www.ClinicalTrials.gov,](http://www.ClinicalTrials.gov) as required by U.S. Law. This Web site will not include information that can identify you. At most, the Web site will include a summary of the results. You can search this Web site at any time.

The study sponsor shall use the information collected during this study for research purposes only, which may include the following:

- Reviewing the safety or effectiveness of the study device and other products or therapies;
- Conducting performance reviews of the study device or retrospective reviews of the study or the study data;
- Evaluating other products or therapies for patients;
- Developing a better understanding of disease; or

Improving the design and efficacy of future clinical trials.

- **QUESTIONS**

Contact [Name] at [Phone number(s) (24-hour number required)] for any of the following reasons:

- if you have any questions about this study or your part in it,
- if you feel you have had a research-related injury, or
- if you have questions, concerns or complaints about the research.

If you have questions about your rights as a research subject or if you have questions, concerns or complaints about the research, you may contact the study IRB:

Western Institutional Review Board^®^ (WIRB^®^)

1019 39th Avenue SE Suite 120

Puyallup, Washington 98374-2115

Telephone: 1-800-562-4789 or 360-252-2500

E-mail: Help@wirb.com

The IRB is a group of people who independently review research.

The IRB will not be able to answer some study-specific questions, such as questions about appointment times. However, you may contact the IRB if the research staff cannot be reached or if you wish to talk to someone other than the research staff.

Do not sign this consent form unless you have had a chance to ask questions and have gotten satisfactory answers.

If you agree to be in this study, you will receive a signed and dated copy of this consent form for your records.

**CONSENT**

- I have read all parts of this Subject Informed Consent Form.
- I have had the opportunity to ask questions about any part that I have not understood and have received satisfactory answers.
- I was informed about the study plan, study requirements and about my rights.
- Once my study doctor and I sign this form, I will receive a signed copy of this entire document.
- By signing this form, I agree to participate in this study.
- I authorize Nevro Corp. personnel to contact me by phone, e-mail, and/or text message (pursuant to my selection(s)) above, and I understand that my standard message and data rates will apply. I also understand the risks of allowing my health information to be sent by e-mail or text message.
- I understand my participation is voluntary and I can leave the study at any time without losing my rights to other treatments I may need.
- I may also be withdrawn from the study at any time with or without my consent, and possibly be asked to have the device taken out.
- I authorize the release of my medical records for research or regulatory purposes to the sponsor, the FDA, the DHHS, governmental agencies in other countries, and the IRB.

By signing this consent form, I have not given up any of my legal rights.

Subject Name (printed)

**Consent Signature:**

__________________

Subject Signature Date

Attestation Statement:

I confirm that the research study was thoroughly explained to the subject. I reviewed the consent form with the subject and answered the subject’s questions. The subject appeared to have understood the information and was able to answer the following questions correctly:

1. What is the purpose of this study?
2. If you decide to be in the study, what will you be asked to do?
3. What is the possible benefit of participating in this study?
4. What are the possible risks of participating in this study?
5. If you decide not to participate in this study, what options do you have?
6. Will participating in this study cost you anything? If so, what will you have to pay for?
7. Do you have to be in this study?
8. If you decide to be in the study, can you leave the study when you want to?

__________________

Printed Name of Person Conducting the Position

Informed Consent Discussion

__________________

Signature of Person Conducting the Date

Informed Consent Discussion
